# An automated and high-throughput data processing workflow for PFAS identification in biota by direct infusion ultra-high resolution mass spectrometry

**DOI:** 10.1007/s00216-024-05426-2

**Published:** 2024-08-01

**Authors:** Silvia Dudášová, Johann Wurz, Urs Berger, Thorsten Reemtsma, Qiuguo Fu, Oliver J. Lechtenfeld

**Affiliations:** 1 Department of Environmental Analytical Chemistry, Helmholtz Centre for Environmental Research – UFZ, Permoserstrasse 15, 04318 Leipzig, Germany; 2 Institute for Analytical Chemistry, University of Leipzig, Linnéstrasse 3, 04103 Leipzig, Germany; 3 ProVIS – Centre for Chemical Microscopy, Helmholtz Centre for Environmental Research – UFZ, Permoserstrasse 15, 04318 Leipzig, Germany; 4 Present Address: Laboratory of Clinical Biochemistry and Metabolism, Department of General Pediatrics, Adolescent Medicine and Neonatology, Faculty of Medicine, University of Freiburg, 79106 Freiburg, Germany

**Keywords:** Ultra-high resolution mass spectrometry, Suspect screening, PFAS, Automated data processing, KNIME

## Abstract

The increasing recognition of the health impacts from human exposure to per- and polyfluorinated alkyl substances (PFAS) has surged the need for sophisticated analytical techniques and advanced data analyses, especially for assessing exposure by food of animal origin. Despite the existence of nearly 15,000 PFAS listed in the CompTox chemicals dashboard by the US Environmental Protection Agency, conventional monitoring and suspect screening methods often fall short, covering only a fraction of these substances. This study introduces an innovative automated data processing workflow, named *PFlow*, for identifying PFAS in environmental samples using direct infusion Fourier transform ion cyclotron resonance mass spectrometry (DI-FT-ICR MS). *PFlow*’s validation on a bream liver sample, representative of low-concentration biota, involves data pre-processing, annotation of PFAS based on their precursor masses, and verification through isotopologues. Notably, *PFlow* annotated 17 PFAS absent in the comprehensive targeted approach and tentatively identified an additional 53 compounds, thereby demonstrating its efficiency in enhancing PFAS detection coverage. From an initial dataset of 30,332 distinct *m/z* values, *PFlow* thoroughly narrowed down the candidates to 84 potential PFAS compounds, utilizing precise mass measurements and chemical logic criteria, underscoring its potential in advancing our understanding of PFAS prevalence and of human exposure.

**Graphical abstract:**

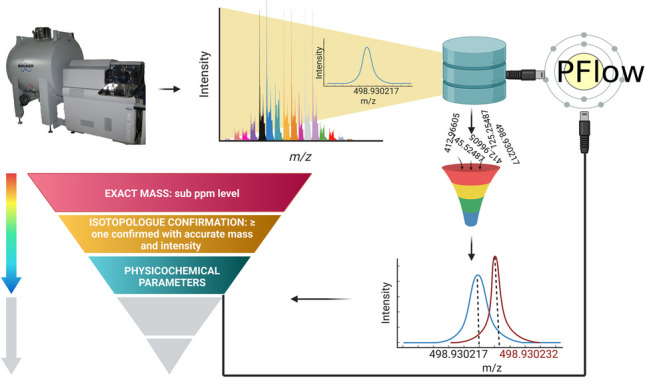

**Supplementary Information:**

The online version contains supplementary material available at 10.1007/s00216-024-05426-2.

## Introduction

Per- and polyfluorinated alkyl substances (PFAS) represent a class of anthropogenic compounds produced since the mid-twentieth century [1]. Due to their amphiphobic character and stable C-F bond, they are used in various industrial and consumer products and processes [1–3]. By the beginning of the 2000s, they gained worldwide attention as long-chain PFAS were detected globally, which raised concerns about their environmental stability and bioaccumulation in biota [4–7]. Since then, numerous long-chain PFAS were either phased out or reduced, initiating the production of substitutes [8–10]. Epidemiological studies have associated specific PFAS exposure with diverse health effects, including immune and thyroid disruption, liver disease, reproductive and developmental problems, and cancer, among other adverse outcomes [11–15]. While exposure typically encompasses various PFAS, there is limited knowledge about the exposure to new PFAS substitutes [11, 16]. As of 2021, 531 PFAS have been registered under Registration, Evaluation, Authorization, and Restriction of Chemicals (REACH) in the European Union (EU), with an estimated annual release of 75,000 metric tons into the environment in Europe [17, 18]. Five national authorities jointly proposed to the European Chemicals Agency (ECHA) to restrict all PFAS under REACH in Europe, aiming to mitigate PFAS pollution and its associated environmental and health risks [19].

The identification and characterization of PFAS pose significant challenges due to their chemical diversity and the dynamic nature of development and manufacturing processes [20]. Conceptually, there is no universal analytical technique capable of measuring the entire range of PFAS. The choice of analytical instrument is contingent on various factors, considering the trade-offs between sensitivity, selectivity, and the ability to provide molecular information. Techniques such as combustion ion chromatography (CIC), particle-induced gamma emission (PIGE), inductively coupled plasma mass spectrometry (ICP-MS), and the total oxidizable precursor assay (TOPA), provide a collective assessment of the totality of (organic) fluorine, or of PFAS and their so-called precursors, without identifying the individual compounds. While coupling separation methods with mass spectrometers is a potent strategy, especially beneficial in environmental monitoring and regulatory compliance, it tends to target specific PFAS groups, utilizing predefined methods and standards for identification and quantification and does not account for unknown or unexpected chemicals [21–26].

Suspect and non-target screening diverge from traditional target-based analytical methodologies by not limiting its focus solely to predefined PFAS compounds for analysis. Instead, the approach employs high-resolution mass spectrometry (HRMS) techniques. This broader strategy enables the identification of a wide array of PFAS compounds, including those not previously considered or known, by leveraging the comprehensive scanning capabilities of HRMS to detect molecular ions and their fragments. However, such approaches require a balance between the need for comprehensive screening, which produces extensive datasets for each sample, and the practical constrains of data management and analysis to reduce the number of features. To minimize the risk of false-positive assignments, several strategies, including blank subtraction, signal intensity thresholding, and mass defect filtration, can be employed to streamline data analysis and prioritize PFAS compounds [27–30]. Several approaches were developed and implemented to identify PFAS candidates, designating them for further analysis. Previously employed “in-source fragmentation flagging” effectively identified PFAS compounds through unique in-source fragmentation ions, but its reliance on specific ions can cause misidentifications of non-PFAS compounds that produce similar ions or PFAS compounds might be overlooked if they behave differently under varying conditions [31–33]. This issue becomes pronounced in complex environmental samples, where many compounds share similar fragmentation patterns. Moreover, the application of specific isotopologue distributions can enhance the identification of PFAS. For instance, Zweigle et al*.* applied a carbon-normalized mass defect plot to prioritize features based on the separation between fluorinated compounds and matrix-related interferences. Although this technique proved effective for perfluorinated compounds, it showed limitation in efficiently separating polyfluorinated compounds with a higher hydrogen content, thus increasing the potential for false positives [34, 35].

Ultra-high resolution Fourier transform ion cyclotron resonance mass spectrometry (FT-ICR MS) is the highest-performing mass analytical technique primarily used to characterize complex mixtures [36]. As a result of its ultra-high resolving power (10^6^ @ *m/z* 400), and low mass error (< 100 ppb), this method facilitates the unequivocal assignment of the elemental composition for thousands of compounds without the necessity for prior chromatographic separation or fractionation [37, 38]. Recently, Young and Pica demonstrated how the ultra-high resolving power and mass accuracy of 21 T FT-ICR MS enabled identifying 163 known and 134 novel PFAS in aqueous film-forming foam (AFFF) mixtures with non-target screening (NTS) using Kendrick-analogous mass difference analysis [39]. This approach relies on accurate mass measurement that detects isotopologues and recognizes homologs based on their molecular ions, greatly reducing the number of candidates generated during molecular formula assignment. Similarly, D’Agostino and Mabury conducted direct infusion (DI-) FT-ICR MS to study AFFF and commercial surfactants concentrate. The use of FT-ICR MS was crucial for resolving two signals with a mass difference of only 0.059 Da [40]. Notably, as of October 2022, the CompTox database contains 6604 neutral molecules, among which there are 5068 pairs of molecules distinguished by a monoisotopic mass difference of less than 0.059 Da.

Employing ultra-high resolution (UHR) MS without the need for prior separation offers a significant advantage in detecting PFAS directly from samples, bypassing the limitations often associated with separation techniques. This approach is particularly suitable for high-throughput measurements, as it enables fast data analysis and yields a reduced number of potential candidates. Operating within the realm of high mass accuracy facilitates a narrow and focused preselection of candidates, streamlining the identification process by minimizing the pool of potential matches. These preselected candidates can then undergo further verification through detailed isotopic analysis, leveraging the distinct isotopic patterns characteristic of fluorinated compounds to confirm their presence, thus optimizing both the speed and accuracy of the screening process. However, the application of this technique for detecting PFAS in environmental or biota samples, with concentration potentially as low as 0.01 µg/kg^−1^ in environmental or biota samples has yet to be demonstrated.

In this study, we address three key points: Firstly, previous applications of FT-ICR mass spectrometry focused on highly concentrated AFFF samples, containing a substantial amount of PFAS compounds. Here, we aim to demonstrate the use of DI-FT-ICR MS for PFAS identification in biota samples and present an analytical method using mass spectral stitching to increase the sensitivity for low-concentrated analytes. Secondly, we developed an open-access, interactive data processing workflow (called *PFlow*) tailored for automated, high-throughput PFAS suspect screening analysis, simplifying data evaluation, visualization, and exploration. Lastly, we present a complementary workflow for suspect list curation (called *PFAS:SL*), aiming to standardize data types and minimize the risk of excluding compounds during the data evaluation process. Both workflows are openly accessible, with detailed information on functionalities and implementations provided. This contribution aims to provide the community with advanced tools for PFAS analysis, fostering transparency, reproducibility, and collaborative research endeavors.

## Materials and methods

### Samples and measurements

#### Samples

The bream liver sample *(lat. Abramis brama)* originates from the river Rhine (Bimmen, Germany), collected in 2001 as part of the German Environmental Specimen Bank monitoring program of the federal German Environment Agency (UBA) and was previously analyzed in the FLUORBANK project [41].

#### Sample preparation

Prior to extraction, the bream liver sample was stored in a polypropylene (PP) vial at −18 °C. Approximately 1 g (wet weight) of sample was wetted with 3 mL acetonitrile (ACN). After solvent evaporation, samples were extracted with 5 mL ACN, vortex-mixed vigorously (Heidolph, REAX 2000), ultrasonicated for 15 min at 25 °C (Bandelin, SONOREX RK 510), and centrifuged (Eppendorf 5804) for 5 min at 2000 rpm. The extraction was repeated three times.

Under nitrogen atmosphere, the extract was concentrated to approximately 1 mL, and subsequently transferred to a 2-mL centrifuge tube containing 20 mg graphitized carbon (ENVI-Carb, Superclean, 120/400 mesh, Supelco, Sigma-Aldrich, Bellefonte, USA) and 50 µL glacial acetic acid (AcOH). The tube was vigorously vortex-mixed and centrifuged (10 min at 10,000 rpm). The final extract was treated with 500 µL of 4 mM ammonium acetate (NH4AC) and stored at −18 °C. Before analysis, all extracts were filtered through a 0.2-µm RC4 filter (Minisart, PP-housing, Sartorius, Stonehouse, UK) into PP autosampler vials.

#### LC-HRMS method and data analysis

The HRMS screening was performed on a quadrupole time-of-flight mass spectrometer (qTOF) (Xevo G2-XS TOF, Waters, Milford, Germany) in continuum mass spectrometry elevated energy data acquisition method, which enhances sensitivity by capturing both precursor and fragment ions in a single run, utilizing all ions for comprehensive data collection. The analyzer mode was set to sensitivity. A 15 to 45 eV ramp was applied as high collision energy (CE), and the mass window from 50 to 1200 Da was scanned in continuum mode with a scan time of 0.15 s without CE acquisition.

An injection volume of 10 µL was separated on an Acquity UPLC BEH Shield RP18 column (100 × 2.1, 1.7 µm, Waters) at a flow rate of 0.35 mL/min and a column temperature of 45 °C. The gradient program started with 90% solvent A (2 mM ammonium acetate in water/methanol, 95/5, v/v) and 10% solvent B (2 mM ammonium acetate in water/methanol/acetonitrile, 5/75/20, v/v/v). After 1.5 min, the proportion of solvent B was ramped to 65%, after 4.5 min to 80% and after 8.25 min to 99.9%. This condition was held for 2.75 min before returning to the initial conditions. The total run time was 15 min. The measurements were conducted in ESI-negative mode, employing N_2_ as desolvation and cone gas (600 and 150 L/hr, respectively). The capillary voltage was set at 0.8 kV, and an optimized desolvation and source temperature of 350 and 120 °C, respectively.

The data analysis was conducted in the TargetLynx (Waters GmbH, Eschborn, Germany), utilizing accurate mass identification (5 ppm) and MS2 confirmation based on the peak shape overlap.

#### FT-ICR MS data acquisition

Samples were measured using a FT-ICR mass spectrometer equipped with a dynamically harmonized analyzer cell (solariX XR, Bruker Daltonik GmbH, Bremen, Germany) and 12 Tesla superconducting magnet (Bruker Biospin, Wissembourg, France). Extracts were diluted 1:100 in a methanol/water mixture (1:1, v/v). A total volume of 100 µL was directly injected via a syringe (type 1725, Hamilton robotics, Bonaduz, Switzerland) at a flow rate of 240 µL per hour into the ESI source (Apollo II, Bruker Daltonics, Billerica, USA). The data was generated in negative ionization mode (capillary voltage = 4.2 kV, nebulizer gas pressure = 1.0 bar, dry gas temperature = 250 °C, dry gas flow rate = 8.0 L/min). Q-isolation (continuous accumulation of selected ions, CASI) spectra were acquired (detection mass range: 150–2000 m*/z*) by segmenting the mass range using the following *m/z* windows: 150–230, 230–270, 270–300 and from 300 segmented by 100 Da up to 1000, and 1000 to 2000 Da with 1000 ms ion accumulation time. For the bream liver sample, 11 CASI spectra were acquired in this manner, and each spectrum had between 2500 and 6500 detected signals (S/N ≥ 4). Mass spectra were internally calibrated using a list of lipids and their derivatives (between 87 and 1572 m*/z*, *n* = 473) resulting in mass accuracy < 0.5 ppm (*n* = 71).

#### FT-ICR MS data pre-processing

Calibrated data were uploaded to *PFlow* from the local file system in *CSV* (comma separated values) format and were subsequently evaluated. Several fluorinated compounds, such as perfluoroalkyl sulfonates, have a molecular weight that lies at the edge of a CASI mass window. For example, PFOS with a mass of 498.93022 Da was included in the CASI window ranging between 400 and 500 m*/z* but leads to ^18^O isotopologue detection (500.93446 Da) in the subsequent mass window which would cause low similarity score because of a missing signal. For this reason, each spectrum was filtrated according to the CASI windows with the overlap of ± 5 Da retaining duplicated molecular formulas in the final candidate list that were then filtrated out at the beginning of the data evaluation step described in the “Suspect filtering and verification” section.

### PFAS suspect screening

#### Compilation of the PFAS suspect list

Rationale and concept: In recent years, many PFAS suspect lists have been compiled and made publicly available, with the aim to simplify the search for relevant substances in mass spectrometry screenings. However, incorporation of suspect lists into automated data processing workflows requires a uniform machine-readable format to provide consistent and efficient chemical identification, which becomes crucial while compiling lists from different origins. Here, inconsistencies can result in gaps in compound coverage, potentially leading to the oversight of relevant substances during analysis. Secondly, suspect lists are often created independently, and there may be limited cross-validation between different lists. This lack of validation can lead to uncertainties regarding the accuracy and reliability of identified compounds. Additionally, the presence of compounds in different forms (e.g., salts, charged molecules) complicates the identification process. Mass spectrometry relies on accurate mass measurements for compound identification. If the compound is listed in a different (chemical) form, it may be challenging to match the observed data to the correct compound, impacting the reliability of the identified substances. Lastly, the inclusion of isotopologues in a database allow for more accurate mass calculations and assists in verifying the elemental composition of a compound.

Procedures and calculations: The CompTox Chemicals Dashboard is a comprehensive collection of existing PFAS containing 12,034 entries (downloaded: 2022-10-10 from https://comptox.epa.gov/dashboard/chemical-lists/PFASMASTER) [42, 43]. A sequence of operations was performed on the CompTox PFAS list to facilitate its use with ultra-high resolution mass spectral data, resulting in a new comprehensive PFAS suspect list, which we call *PFAS:SL* (see Electronic Supplementary Material Section S1-2, Figure S1B). The procedures comprised the following steps: (i) the recalculation of monoisotopic masses to ensure consistency across the database, (ii) the extraction of information from existing chemical identifiers after molecular formula generation to complete missing InChI (International Chemical Identifier) keys and SMILES (Simplified Molecular Input Line Entry System) for certain compounds. (iii) the computation of isotopologue masses for selected isotopes (C, S, O, Br, Cl, Si) and their linkage to the monoisotopic mass based on their unique IDs. The calculation of isotopologue masses was limited to the most abundant isotopes. This decision was based on the rationale that including only the masses of the most abundant isotopologues in the database enhances the likelihood of their detection in measurements. Low-abundance isotopes, present in smaller quantities, might pose challenges in terms of detection or contribute minimally to the overall signal in analytical measurements. Isotopologues are commonly used to facilitate elemental composition calculation from accurate masses and isotope intensities. For *PFlow*, we also use isotopologues for suspect matching confirmation and to exclude potential false positive matches. (iv) The computation and storage of elemental ratios (F/C, H/C, H + F/C, O/C, DBE and DBE-O, element count, mass defect) in *PFAS:SL*, aiming to offer an overview of the covered chemical space. Altogether, *PFAS:SL* contains 34,658 entries, of which 10,905 correspond to monoisotopic masses (Figure S2-5).

The workflow provides users with the ability to create their own suspect list for use in *PFlow*. To initiate this process, input files have to be prepared in the *CSV* format. This file must include a column labeled “Molecular formula” as the first column. In cases where the molecule exists in salt form, an additional chemical identifier such as InChI key, IUPAC (International Union of Pure and Applied Chemistry) name, or SMILES is required for mass recalculation; otherwise, the workflow will treat the substance as being in its neutral form. A template is provided for users’ convenience.

#### Suspect matching

Rationale and concept: To initiate the suspect matching, every *PFAS:SL* entry was recalculated to the deprotonated/negatively charged accurate mass that involved adjusting the mass value by either removing a proton from neutral molecules or removing a counter-cation (from salts) to reflect the negatively charged state. Some compounds in the CompTox database do not contain hydrogen and are therefore unlikely to be ionized in negative ionization mode. These were included in the suspect list and used in subsequent screening. If these compounds appeared in the matched suspect list, we applied the exclusion method—pKa value filtering—to remove them, as they likely lack acidic ionization sites (e.g., perfluorohexane). Given that our suspect list contains structural information, we can predict pKa values. However, we cannot guarantee that some compounds do not exist in different isomeric forms that could enable their detection in negative ionization mode. The concept is described in the “Suspect filtering and verification” section.

The suspect matching follows the basic principle of mass pair aligning between the measured signals (*m/z* values) and the PFAS suspect list (here: *PFAS:SL*) to assign to the fluorinated compounds monoisotopic masses in measured spectra. For this purpose, we implemented a distance-based method, Euclidean distance, where each *PFAS:SL* entry was searched against measured *m/z* using specified neighbor criteria (neighbor count: 10, mass error ≤ 1.5 ppm). The described process involves selecting ten monoisotopic masses for each *PFAS:SL* entry value and forming pairs with each of them. The subsequent calculation of mass accuracy for these pairs helps assess the accuracy of the mass measurements in the context of the expected monoisotopic masses. If the mass difference between molecules in the *PFAS:SL* was lower than the specified mass error, e.g., C_11_H_6_Cl_2_F_6_N_2_ (349.98122 Da) and C_6_H_2_F_12_O_3_ (349.98123 Da) with a mass difference of 0.03 ppm, both *candidates* would be retained and treated as distinct annotation of the same measured signal (*candidate* annotations). For detected monoisotopic masses, corresponding isotopologues were used to calculate abundance ratios and compared to peak intensities of isotopologue *m/z* values within the measured spectra. By evaluating the alignment between the expected and observed isotopic patterns, we can verify the accuracy of mass measurements and gain confidence in the assigned molecular formula. For this purpose, we also used Euclidean distance calculations to quantify the dissimilarity between observed and theoretical isotopic distributions. All *candidates* that could be verified by isotopologue peak intensity obtained a similarity score and were classified as *L-4 candidates*; the remainder of *candidates* that could not be confirmed by isotopologue peak intensity were classified as *L-5 candidates* (i.e., annotated by accurate mass but without isotopologue confirmation).

Procedures and calculations*:* Within the workflow, users can choose from a list of ion-forming adducts based on the measurement mode and their specific requirements. All measurements in this study were conducted in the negative ESI mode, and we assume deprotonation as the major ion formation process ([M-H^+^]^−^). Therefore, in the case of neutral molecules, mass deconvolution was performed by subtracting of a single proton mass *(accurate mass* = *m/z—1.007277 (mass of H*^+^*))*, while negatively charged molecules were excluded from this step. For molecules in the form of salts, the counter-ion was stripped from the molecule, and the mass was recalculated and utilized in the subsequent steps. While the current method focuses on anionic PFAS, it is important to note its limitations concerning neutral and cationic compounds that cannot be ionized in negative ESI mode. However, this limitation could be addressed by measuring samples in different modes that target these compounds.

The isotope scoring method involves the comparison of the observed isotopic distribution of a measured compound with the theoretical distribution based on its elemental composition. For this purpose, we employed the following calculations:

The Euclidean distance is defined as the square root of the sum of the squares of the difference between corresponding coordinates (p_i_, q_i_) of the two vectors in the Euclidean space [44, 45]. If *p* = (*p*_1_, *p*_2_) and *q* = (*q*_1_, *q*_2_) are two points in the plane, the distance is given by Eq. 1:

1 $$\begin{array}{c}{d}_{p,q}=\sqrt{{\left({p}_{1},{q}_{1}\right)}^{2}+{\left({p}_{2},{q}_{2}\right)}^{2}}\\ =\sqrt{{\sum }_{i=1}^{n}{\left({p}_{i},{q}_{i}\right)}^{2}}\end{array}$$

Firstly, we calculated the Euclidean distance between two variables: signal intensity (*y*-axis) and mass to charge ratio (*x*-axis). Due to the scale differences between *m/z* and signal intensity, we expressed mass deviation in ppm and the signal intensity as relative percentage differences to avoid mixed attributes in the computation of distance. Since we were interested in the similarity between two isotopologues (measured and theoretical), it is essential to convert distant metric into similarity metric by Eq. 2 to obtain a value between 0 and 1 (0 = dissimilar, 1 = similar).

2 $$Similarity=\frac{1}{1+d\left ({p},{q}\right)}$$

Secondly, as the isotopologue intensity is proportional to the relative abundance of the isotopes, we weighted similarity score according to the isotope contribution that is proportional to the signal intensity. So, high intensity signals contribute more to the weighted mean than do signals with a low intensity according to Eq. 3:

3 $$W=\frac{{\sum }_{i=1}^{n}{w}_{i}{x}_{i}}{{\sum }_{i=1}{w}_{i}}$$

Lastly, the final score (expressed as %) was calculated according to Eq. 4:

4 $$Isotope\; score=\left({\sum }_{i=1}^{n}{Similarity}_{i}*{W}_{i}\right)*100$$

The algorithm assumes that all matched signals contain measured isotopologues if the simulated intensity is above the minimum measured intensity, so any deviation from the simulated pattern will reduce the similarity score.

#### Suspect filtering and verification

Rationale and concept: Isotope pattern analysis is primarily used in decomposing monoisotopic signals to facilitate determining the best matching elemental composition [46–48]. In case of the PFAS suspect screening, we already computed molecular formulas and their isotopologues (stored in the *PFAS:SL*), but this does not always lead to unequivocal annotations (i.e., multiple PFAS *candidates* for one measured *m/z*). We therefore use a ranking system for the *candidates*, which explicitly considers a set of parameters: the mass error, the physicochemical property pKa, and the quality control annotation (QC) extracted from the CompTox database. Further exclusion of improbable *candidates* can be obtained via a build-in PubChem query for cross-validation. These steps are designed to help the users to rank and ultimately identify the most probable *candidates*.

Procedures and calculations: The initial step in this evaluation is removing duplicate *candidate* annotations in the final *m/z* list, which may occur due to the overlap of mass windows or because the *candidate* belongs to a parent molecule and its salt. To identify duplicated molecules, where one is stored in the *PFAS:SL* as a salt and another in its neutral form, both annotations will be recognized based on their “charged mass,” which remains consistent for both molecules. In this case, the *candidate* with the higher S/N ratio of its corresponding mass peak is retained.

Mass errors in UHRMS are indicative of the accuracy of molecular identification, assuming optimal mass spectral calibration. Consequently, *candidates* exhibiting lower mass errors are frequently regarded as true matches, implying that the observed mass closely corresponds to the theoretical mass of a particular compound. However, several factors influence the measurement’s mass accuracy and resolving power and may obscure this “best fit” approach [49]. We included additional physicochemical parameters of the fluorinated compounds in *PFAS:SL* to increase the confidence of the *candidate* annotations and rule out implausible *candidates*.

The pKa value is a fundamental property in chemistry that quantifies the acid dissociation constant (Ka) of a substance, essentially measuring the strength of an acid in solution, and is crucial for predicting how substance will interact in various pH environments [50]. In a recent study, the pKa value was suggested as important molecular parameter in predicting ionization efficiency (IE) in ESI-MS [51]. This was demonstrated for several compound classes (e.g., phenols and benzoic acids), revealing that the degree of ionization of the molecule and the charge delocalization in the anion are the most significant parameters in describing IE in ESI-(-) [51, 52]. In our study, the pKa value was utilized to refine our candidate list, prioritizing molecules with acidic pKa while excluding those without an acidic functional group. All pKa values were generated in the ChemAxon predictor (available at: https://chemaxon.com/calculators-and-predictors) [53].

The CompTox Dashboard employs the classification of confidence levels in chemical identification ranging from level 5 to level 1, denoting varying degrees of confidence used here as compound quality control (QC). Starting with level 5, identifications are programmatically curated from either ACToR or PubChem, representing the initial stage with low confidence and reliance on a single public source. Progressing to level 4, the process involves programmatically curated data from ChemID, ensuring that unique chemical identifiers exhibit no conflicts within PubChem, thereby increasing the reliability of the data. At level 3, the curation becomes more stringent, with programmatically curated data sourced from high-quality EPA sources, further ensuring that unique chemical identifiers remain consistent and conflict-free across both ChemID and PubChem databases. Level 2 marks a significant transition to expert curation, where unique chemical identifiers are derived from multiple sources, enhancing the accuracy and reliability of the chemical identification. Finally, level 1 represents the highest confidence in chemical identification, with expert curation ensuring accuracy and consistency of unique chemical identifiers, establishing a solid foundation for scientific analysis and research [42, 43].

Considering the three specified variables, the table’s final candidates are organized according to the normalized values of mass accuracy, QC level, and pKa, with a preference for lower values in each category. Each variable is normalized on a 0 to 1 scale, where 0 signifies the most desirable outcome and 1 the least. Specifically, the mass accuracy is normalized such that the lowest error corresponds to 0 and the highest to 1. The QC levels, which are discrete and ranging from 1 (highest confidence) to 5 (lowest confidence), are directly normalized, maintaining the preference for lower numbers. For the pKa values, which prioritize more acidic substances (lower values, including negatives), normalization is conducted so that the lowest observed pKa maps to 0, emphasizing the selection of strong acids. A composite score for each candidate is then calculated by averaging these normalized values, ensuring that lower scores highlight candidates more closely aligning with desired criteria.

Lastly, mass accuracy and isotope pattern matching are central when assigning candidates from DI data, even with the support of ultra-high mass resolution. However, verifying *candidates* with very low peak intensity through isotopologues can be prone to errors or become even impossible, e.g., if the peak intensity is too low to detect isotopologues above the minimum allowed signal-to-noise threshold. To address this issue and support in the identification of fluorinated compounds of low abundance, we have added a function that extracts all potential compounds (i.e., their distinct molecular formulas) from PubChem for a particular *candidate’s m/z*. However, this step is optional.

## Results and discussion

### Automated suspect screening with *PFlow*

In order to automate PFAS suspect screening from UHRMS data, the comprehensive PFAS suspect list, *PFAS:SL*, was combined with the formula validation approaches into a data processing workflow called *PFlow*. *PFlow* is an automated data processing workflow designed for processing DI-UHRMS data acquired from FT-ICR MS. While it can handle high-resolution mass spectrometric data, it is not recommended for low-resolution data, as this can lead to multiple molecular formulas for the same signal, affecting overall accuracy. It is implemented in the open-source software KNIME Analytics Platform (version 4.7.5). With a combination of R scripts, Python scripts, and native KNIME nodes, it performs molecular mass matching and validates them with heavy isotopes (C, S, O, Cl, Br, Si). The workflow is available online with free access and can be downloaded from 10.5281/zenodo.11633375.

**Fig. 1:**
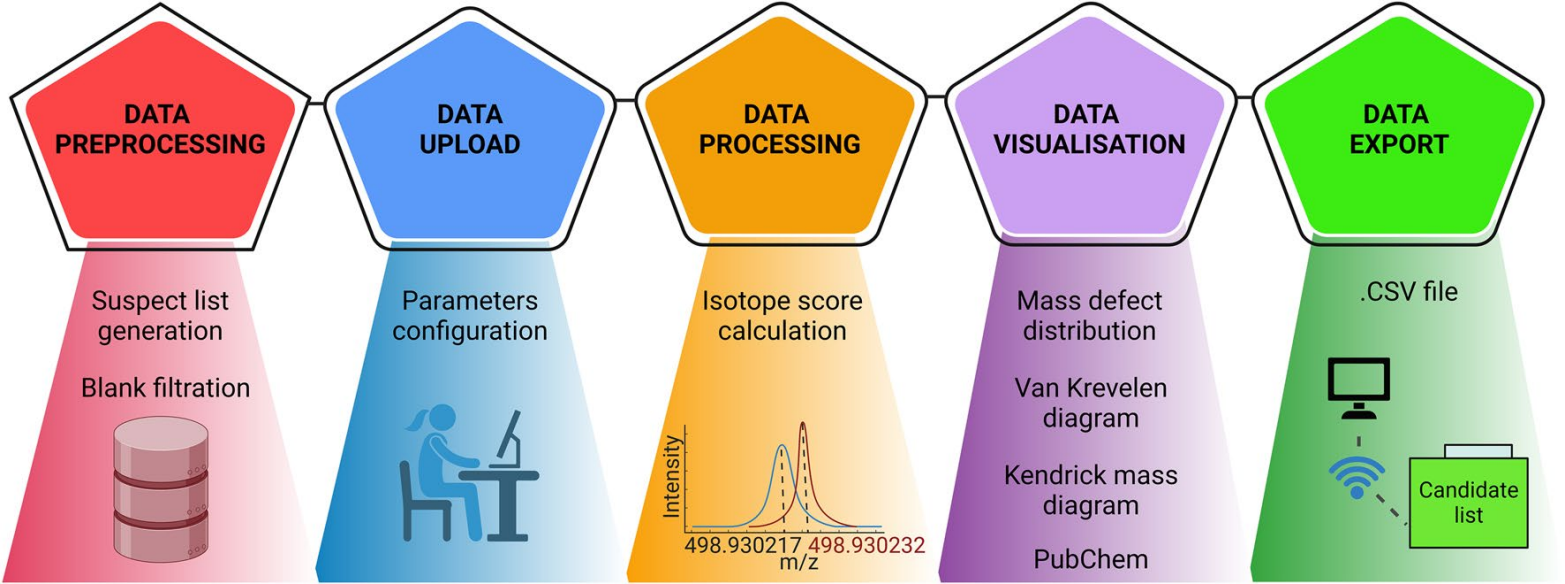
Overview of the main *PFlow* (PFAS suspect screening workflow) data processing steps. Each step represents one major task described in the main text

*PFlow* is divided into five main steps: (1) pre-processing, (2) configuration, (3) suspect matching, (4) visualization, and (5) data export (Fig. 1).

1) Data pre-processing is the initial step in the data analysis pipeline, aimed at enhancing the quality and reliability of the data. The pre-processing step includes two (optional) steps: blank filtering and compilation of a PFAS suspect list. The blank filtering step identifies potential blank *m/z* values within the mass accuracy threshold. The user may apply a filtering step to remove flagged data points based on the intensity ratio between blank and sample data sets. The second option is the compilation of the PFAS suspect list. The user can use the default *PFAS:SL* or use the suspect list generation workflow (as described above) to compile an own suspect list for *PFlow* suspect screening.

2) The initial step in the data processing workflow requires uploading data and configuring parameters. The configuration process is straightforward and user-friendly, providing an intuitive experience that keeps the user well-informed about the parameters necessary for the execution of the workflow. Parameter settings used for data filtration based on measured (S/N, signal intensity threshold) or calculated values (ppm error, similarity score, mass defect) can also be set to refine the candidate list: a higher S/N threshold may be required for samples with a high PFAS content, while a more sensitive approach may be used to detect transformation products at low concentrations (e.g., in biota samples). *PFlow* offers a clear and concise way to process data as a single or multiple *CSV* files executed sequentially from the same local directory. Once the user’s parameters are specified, the data processing is automated upon the workflow execution. The configuration enables users to take control of their data processing and analysis, fostering adaptability, and efficiency.

3) Suspect matching involves *m/z* deconvolution, suspect matching with the *PFAS:SL*, and score calculation. Each step is detailed in the “PFAS suspect screening” section.

4) Finally, *PFlow* provides an interactive user view promoting relation and pattern exploration within the data set through Kendrick mass defect plot (KMD), mass defect distribution, and the elemental ratio plots. At this stage, the opportunity to query the PubChem database is provided by selecting specific *m/z* from the candidate list and retrieving all possible molecular formulas from PubChem within the designated mass error window. This integration is initiated by user-defined queries embedded within the workflow, enabling real-time access to external database based on specific criteria. Such a strategy enhances the capabilities of *PFlow* by allowing users to explore additional molecules that may exist in the sample.

5) The export functionality is included as a terminal step in the workflow, ensuring that users can easily transition from data processing to results interpretation and further analysis. *CSV* is chosen as the export format due to its simplicity and widespread support across various software tools and platforms.

### Identification of PFAS in bream liver via FT-ICR MS

**Fig. 2:**
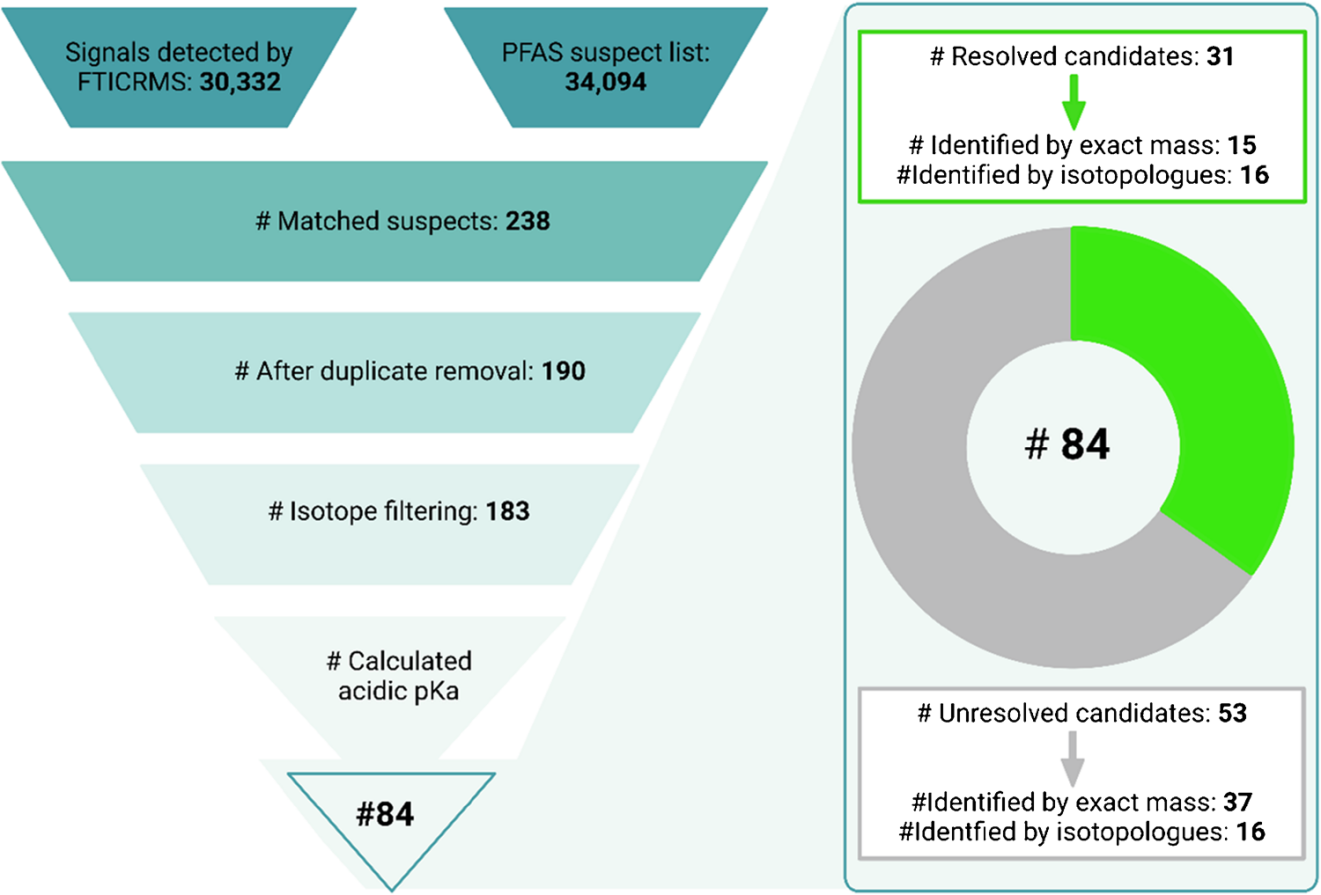
PFAS suspect annotation and elimination of false positives for a bream liver sample with *PFlow*. The schema illustrates the step-by-step reduction in data from the raw FT-ICR MS peak list to the final candidate list. At each step of the process, the count of molecular formulas is indicated, and the validation criteria outlined in the “Methods” section are provided

To demonstrate the applicability of our *PFlow* suspect screening workflow, the extract of a bream liver sample (Rupp et al., 2023) was analyzed by DI-FT-ICR MS and processed by *PFlow*. An overview of the results for each workflow step from this sample is provided in Fig. 2.

First, the mass windows generated in CASI mode are combined to cover the whole mass range from *m/z* 150 to *m/z* 2000. Then, the *PFAS:SL* was searched against 30,332 FT-ICR MS-derived *m/z* values, returning 238 candidates with an allowed mass difference of 1.5 ppm (Fig. 2). Following the candidate matching process, 48 of the initial candidates were identified as duplicate assignments (i.e., occurred twice in the candidate list) and were subsequently eliminated from the selection. Moreover, the list contained suspects with a similarity score of less than 75 (*n* = *7*) which were removed, resulting in 183 remaining candidates (Fig. 2, Figure S5). Based on the pKa filtration step, 64 candidates were excluded due to non-ionizability of the molecule, and an additional 35 were classified as electropositive substances containing basic functional group that can only be ionized in the positive mode (and were thus also excluded for this negative mode measurement). In summary, the final candidate list for the bream liver sample comprises 84 suspects (Fig. 2), with 52 identified through accurate mass and the remaining 32 validated based on isotope pattern. The candidate list was sorted according to their normalized CompTox QC level, mass accuracy, and pKa value, with equal weight given to each of these parameters (Table S1). Notably, at this point, the initial 30,332 distinct *m/z* values were reduced to 84 candidates for PFAS compounds based on accurate mass and chemical plausibility criteria.

### Confirmation of FT-ICR MS-derived PFAS candidates

**Fig. 3:**
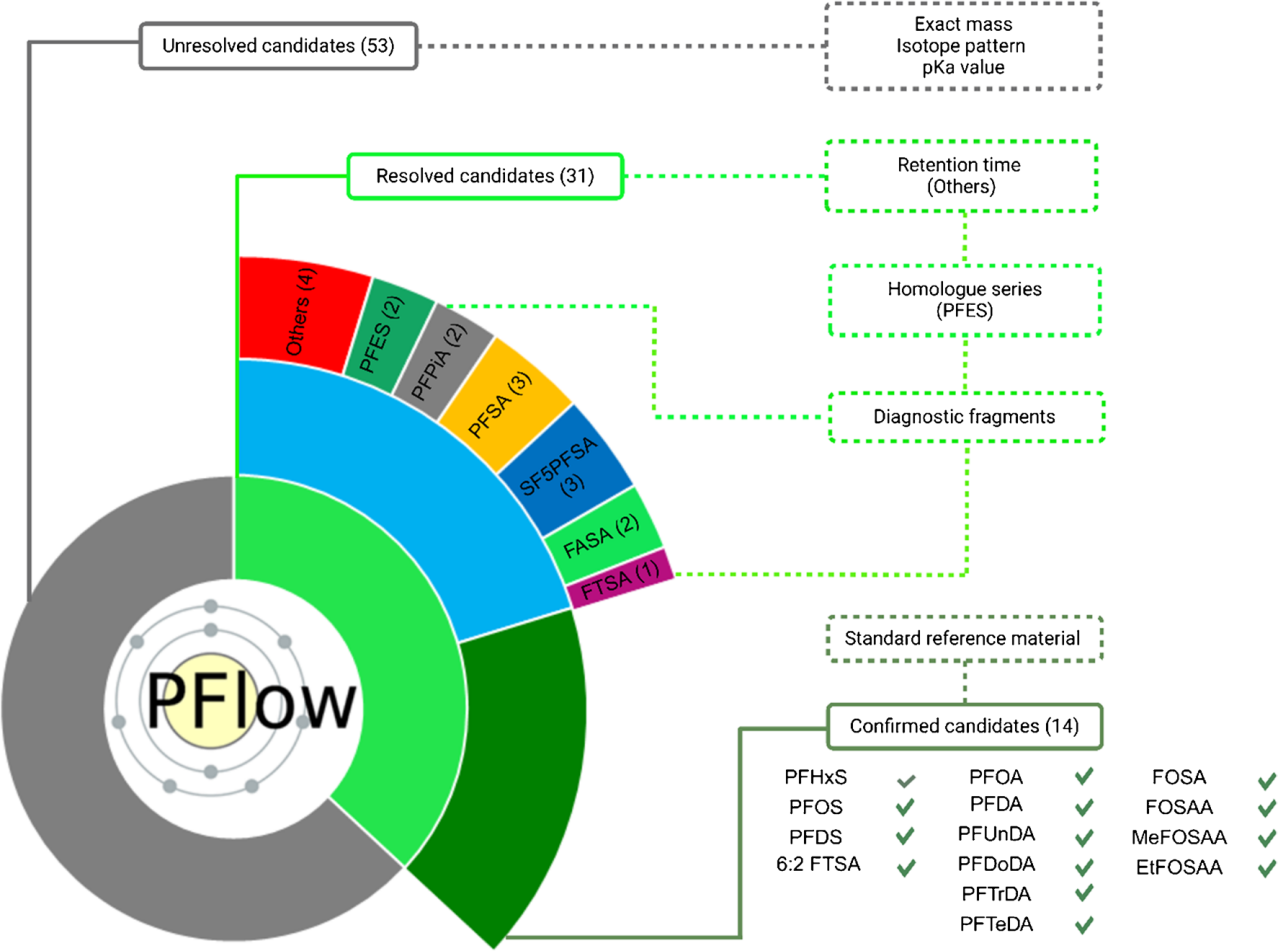
Verification outcome for the PFAS candidates identified with *PFlow*. For a total of 84 candidates, 31 could be verified by LC-HRMS (green segment). Out of those, 14 PFAS were validated by analytical standard (dark green segment) using LC-TQMS, while the remaining 17 candidates were confirmed by diagnostic fragments or homologs of verified PFAS using LC-HRMS

Employing the developed PFAS suspect screening workflow, we tentatively identified 84 candidates in a bream liver sample through a comprehensive suspect screening analysis. The candidate confirmation by LC-qTOF MS then relied on accurate mass, elucidative fragments, and characteristic PFAS features such as homolog grouping, with retention time serving as a secondary confirmation factor for the PFAS group [54]. In the study Rupp et al*.* (manuscript in preparation), a total of 14 candidates were verified through comparison with analytical standards, providing a high degree of confidence in their identification. Additionally, 17 candidates were confirmed at varying confidence levels (CL), ranging from level 2 to level 4, indicating a moderate degree of certainty (Fig. 3). The remaining 53 candidates remained unresolved by means of LC-HRMS.

#### Perfluoroalkyl acids and their derivatives

The most prevalent group in the samples, is associated with perfluorocarboxylic acids (PFCAs) and perfluoroalkane sulfonic acids (PFSAs) exhibiting a uniform fragmentation pattern. Within these two groups, six homologs for each were identified within the confidence level 1–2 (Table S1, Figure S6, S7). Moreover, the perfluorinated phosphinic acids (PFPiAs) specifically PFPiA (C6/C6) and PFPiA (C6/C8) were detected at *m/z* 700.92 Da and 800.92 Da, respectively. These substances were subsequently identified through the neutral loss of the perfluorinated alkyl chain, detected at *m/z* 400.94 Da and 500.94 Da for PFPiA (C6/C6) and PFPiA (C6/C8), respectively, corresponding to C_6_O_2_F_14_P^−^ and C_8_O_2_F_18_P^−^. Confirmation of this group was achieved to CL: 2 (Table S1, Figure S8). Derivatives of PFSAs, pentafluorosulfide perfluoro sulfonates (SF5-PFSAs), were identified (C8-10, where C = No. of carbons). The group was confirmed by fragment ions encompassing the characteristic SO_3_^−^-group and the perfluorinated alkyl chain detected in two homologs with the mass of 279.95, and 379.94, corresponding to SO_3_(CF_2_)_4_^−^, and SO_3_(CF_2_)_6_^−^, respectively (Table S1, Figure S9). The presence of SF5-PFSA aligns with prior findings, as this group has been previously detected in AFFF-impacted groundwater and in aquatic and terrestrial invertebrates [55, 56]. The presence of SF5-PFSA in fish samples is likely attributed to environmental exposure, as these compounds have been detected in water bodies due to their historical use in firefighting activities. In addition to the PFAA group, two PFOS derivatives, Cl-PFOS (CL: 1a), H-PFOS (CL: 4), along with 2-(pentafluoroethyl)-4-(propan-2-yl)pyrimidine-5-carboxylic acid (CL: 4) were identified (Table S1, Figure S10).

#### Perfluoroalkane sulfonamides (FASA)

Perfluorooctane sulfonamide (FOSA, C_8_F_17_SO_2_NH_2_) serves as a common neutral precursor for perfluorooctanesulfonate (PFOS), utilized to repel grease and water in various consumer applications, including food packaging [1]. As a degradation product from sulfonamide-containing precursors, FOSA is widely spread across different media [10, 57, 58]. The perfluorinated alkyl sulfonamides group (FASAs), comprising three homologs, was identified. Two distinctive fragments were associated with this group: the 77.97 Da fragment, corresponding to -NO_2_S, and a fragment with a neutral loss of -HF from the precursor ion. Among these compounds, FBSA, FHxSA were confirmed with CL: 2 and FOSA with CL: 1 (Table S1, Figure S11).

#### Fluorotelomer sulfonic acids (FTSAs)

The fluorotelomer sulfonic acid (FTSA) group, comprising two substances, was identified. Verification was achieved using two specific fragments. The first fragment corresponds to the neutral loss of -HF, while the second fragment involves the loss of -H_2_F_2_ from the precursor ion. The 6:2 FTSA was identified at CL: 1. The 10:2 FTSA was confirmed at CL: 2, demonstrating a criterion for exact mass, retention time, and a characteristic fragmentation pattern (Table S1, Figure S12). Notably, the absence of 8:2 FTSA from the final list of candidates prompted a manual inspection within the FT-ICR MS dataset, especially given its improbable exclusion from the homologous series. Upon manual integration, the signal was indeed detected in the FT-ICR MS data; however, it exhibited a signal-to-noise ratio (S/N) of 1, which did not meet the preset threshold for inclusion among the measured compounds. Therefore, the absence of 8:2 FTSA from the identified candidates by *PFlow* was justified.

#### Perfluorooctane sulfonamido derivatives and EtFOSA-sulfate

Four compounds, namely, FOSAA, Me-FOSAA, Et-FOSAA, and EtFOSA-sulfate, were identified in the sample. The confidence level for this group is at CL: 1 for the first three compounds where EtFOSA-sulfate was identified by a specific fragment with *m/z* of 525.9768, which corresponds to the formula C_10_H_5_F_17_SO_2_N^−^ (CL: 3) (Table S1, Figure S13).

#### Ether-based substances (PFAES)

Two compounds, specifically 1,1,2,2-tetrafluoro-2-[(tridecafluorohexyl)oxy]ethane-1-sulfonic acid and 1,1,2,2,3,3,4,4,5,5,6,6,7,7,8,8-hexadecafluoro-8-(trifluoromethoxy)octane-1-sulfonic acid, were identified. For the latter compound, a single fragment with a mass of 218.94 Da corresponding to C_4_F_9_^−^ was detected (Table S1, Figure S14).

The list of compounds confirmed in our study underscores a diverse exposure to a multitude of PFAS, some of which were previously undetected in liver samples. Notably, compounds like SF5-PFOS and its homologs, which have not been regularly monitored, demonstrated elevated intensity in the liver sample, suggesting their pervasive nature and potential bioaccumulation. The ability to detect such compounds is largely attributed to the utilization of comprehensive and expansive suspect lists, which are fundamental in identifying less common PFAS that might otherwise remain unnoticed. This highlights the imperative need to continuously update and broaden our analytical scope to ensure that emerging and unrecognized PFAS are accounted for, thereby facilitating a more comprehensive understanding of environmental and health risks associated with PFAS exposure.

An overview of the verification process for PFAS suspects (i.e., candidates from *PFlow*) with LC-HRMS is illustrated in Fig. 3. For a detailed list of the annotated PFAS in the bream liver sample, please refer to the SI section (Table S1).

#### Unresolved candidates

The remaining observations not validated with LC-HRMS could be attributed to FT-ICR MS’s higher resolution, sensitivity, and broad mass range, which enhance its ability to detect a wider array of ions in complex mixtures. For instance, two candidates, namely bis(pentacosafluorododecyl)phosphinic acid (*m/z* 1300.8843 Da, rank: 29) and bis(3,3,4,4,5,5,6,6,7,7,8,8,9,9,10,10,11,11,12,12,13,13,14,14,14-pentacosafluorotetradecyl) phosphate (*m/z* 1388.93673 Da, rank: 34), could not be confirmed due to the limited acquisition mass range in LC-qTOF MS (50 to 1200 Da). Additionally, some compounds detected in FT-ICR MS may not be visible in LC-HRMS due to the latter’s reliance on chromatographic separation, which may affect the detection of certain substances, especially those present at very low concentrations or those that do not elute as peak from the chromatographic column under the chosen conditions. A distinct correlation is observable (Table S1) between the molecular rank and the *m/z*. It is apparent that the molecules occupying lower ranks generally exhibit lower *m/z* when compared to those confirmed by LC-HRMS. Furthermore, a substantial portion (*n* = *29*) of the molecules confirmed through LC-HRMS falls within the *m/z* range 300 and 800 Da. These molecules are not only prevalent within this mass range but also tend to receive high rankings based on the variables previously outlined. While this aligns with the initial assumption that the candidates were ranked based on the probability of being true annotations, it does also confirm that high values from our scoring algorithm indicate true positives, i.e., plausible suspect matches. In contrast, there may be manifold reasons why certain candidates remain undetected in LC-HRMS. The prominence of these molecules within this specific *m/z* interval could be indicative of the inherent detection capabilities and biases of the LC-HRMS being employed. Given this observed pattern, one could postulate that molecules with a lower mass may be less likely to be detected by LC-HRMS, which could account for the larger number of unresolved candidates in the lower mass range. The consequence of this could be an underrepresentation of low mass molecules in confirmed results, emphasizing the need to consider the limitations of the analytical methods when interpreting the data.

### Implication of pKa filtration criterion: refining candidates

**Fig. 4:**
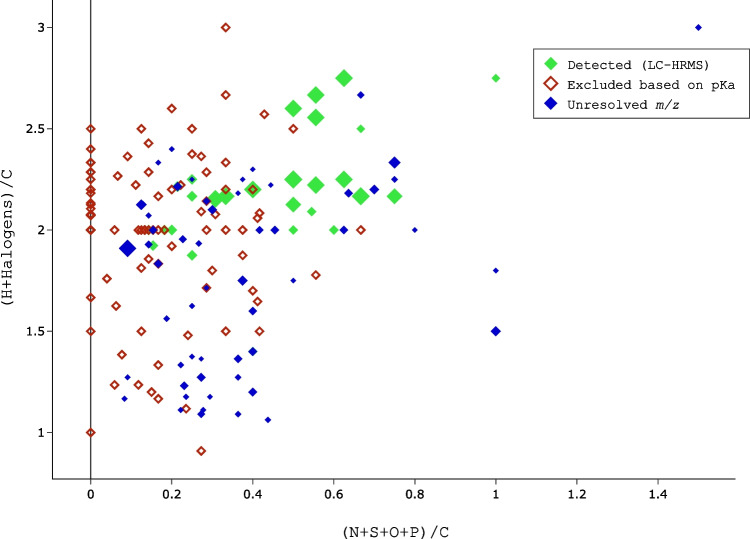
Elemental ratios of candidate PFAS compounds detected by ESI(-)-FT-ICR MS, categorized by three distinct color-coded symbols based on their identification status and pKa property. Candidates are displayed remaining after the initial filtration steps (*n* = 183; cf. Figure 2). Candidates that were detected in an independent LC-HRMS measurement are displayed as green filled symbols (*n* = 31), while blue filled symbols represent unresolved candidates (*n* = 53). Open red symbols denote suspects that were excluded from further consideration based on their pKa (*n* = 99). The size of the diamonds is inverse proportional to the calculated acidic pKa value

Inspection of the distribution of elemental compositions of candidates provides further information about the applicability of the pKa-based exclusion criterion. To this end, the molecular ratios (H + Halogens)/C and (O + S + P + N)/C were used to represent saturation and heteroatom (besides F) content of the candidates, respectively (Fig. 4). Notably, compounds with (O + S + P + N)/C = 0 do not contain any heteroatoms and likely originate from fragments of PFAS formed in the ionization process; these candidates were excluded from further analysis.

Twenty-eight candidates with (H + Halogens)/C >= 2 and (O + S + P + N)/C > 0.1 were detected in subsequent LC-HRMS analysis (Fig. 4, Table S1). Such PFAS compounds are more likely to feature polar functional groups and are often associated with more saturated aliphatic compounds. In contrast, only two candidates with (H + Halogens)/C < 2 were detected by LC-HRMS (Fig. 4), although many candidates corresponded to this class of less saturated, potentially aromatic, and likely more hydrophobic molecules. This indicates that DI-FT-ICR MS can detect PFAS compounds with a much larger elemental composition range compared to the applied chromatographic method. One plausible explanation for this trend can be attributed to the measurement method itself. Direct infusion measurement increases potential for identifying compounds that remain undetected by the LC-MS system, due to either the substance being present in low concentration in the sample or the strong compound affinity towards the mobile or stationary phase in the separation column. These two limitations do not apply to DI-FT-ICR MS. As the sample is directly injected, and only ions from a small mass window are measured, increasing the potential to detect low abundance compounds [59, 60].

To further confirm applicability of the pKa-based exclusion criterion, the Kendrick mass diagram was used to analyze relations between candidates (Figure S15). Two distinct groups emerged, characterized by positive and negative mass defects. The group with positive mass defects consisted of confirmed candidates—notably those with low pKa values—whereas less acidic candidates frequently remain undetected in LC-HRMS. This suggests that a low ionization potential in combination with low abundance render those candidates undetectable in ESI-(-) LC-MS, while they can still be detected with DI-FT-ICR MS in spectral stitching mode (Figure S15).

### Implications for PFAS suspect screening

PFAS suspect screening with FT-ICR MS proves highly beneficial as it initially increases the confidence of molecular formula annotations due to the ultra-high mass resolution and accuracy. The impact of lower accuracy from lower resolution instruments is evident in the number of proposed molecular formulas. For instance, processing the same dataset with a mass tolerance of 5 ppm would have resulted in 685 instead of 238 annotations at 1.5 ppm tolerance.

While suspect screening from DI-FT-ICR MS measurement has clear advantages, it does come with some limitations that require careful consideration. One major limitation of direct infusion is the potential for ionization suppression. While LC reduces sample complexity and helps mitigate ionization suppression, this benefit is not fully realized in our case, where all molecules are introduced simultaneously through the ESI source. However, we use CASI measurement to reduce the complexity of the sample at the detector stage, allowing us to detect molecules that might be suppressed during ionization. Although we have shown that low-concentration molecules can be detected in biota samples, we have not conclusively proven that they won’t be missed in the presence of high concentrations of other analytes. Secondly, in-source fragmentation and adduct formation during the ionization process complicate molecular formula annotation. These may lead to false negatives because a molecular ion is not detected due to fragmentation or to false positives as it is not possible to distinguish between an in-source fragment ion and a molecular ion with the same mass. In addition, isomers cannot be distinguished in DI-FT-ICR MS. However, very low-concentrated isomers (that remain undetected in LC-MS) may still be detected by DI-FT-ICR MS because the signal intensity of all isomers is summed.

To overcome these limitations, we excluded candidates based on their pKa values and elemental compositions, which likely represent in-source fragments (based on known fragmentation patterns for PFAS). The utilization of predicted pKa values appears as a promising tool to exclude unlikely PFAS from suspect screenings, depending on the applied ionization mode. Notably, the exclusion of candidates based on pKa value does not necessarily mean that the mass is a true negative and some PFAS will remain undetected. Conversely, the preselection of candidates based on pKa values does not imply that these PFAS can be verified by LC-HRMS. In other words, candidates that cannot be detected by LC-HRMS are not necessarily false positives.

Further validation with analytical standards is necessary to confirm the final molecular structure of (novel) detected PFAS. *PFlow* aids in this process by providing a sensible preselection of candidates based on a simple scoring to be used in further validation steps.

## Conclusions

We demonstrated the efficiency of the newly developed automated data processing workflow, *PFlow*, to characterize and prioritize PFAS measured by DI-FT-ICR MS. The workflow was validated with a low-PFAS concentration biota sample, where the presence of excessive biogenic material necessitates high mass resolution and low detection limits. The pre-processed data were annotated based on the precursor masses and verified by their isotopologues. Fourteen proposed PFAS in the suspect screening were correctly annotated and confirmed by analytical standard. Furthermore, *PFlow* annotated 31 additional compounds, showing the potential to enhance PFAS coverage compared to the targeted approach. The workflow can be incorporated into expanded data processing pipelines or re-used.

*PFlow* provides an easy and efficient way of data handling to characterize PFAS during suspect screening analysis of DI-UHRMS datasets. With its ability to batch-process ultra-high resolution MS data it paves the way for continuous, high-throughput monitoring of specific masses in the environment, which can collectively contribute to the discovery of PFAS and their transformation products. The presented approach can aid in building a list of commonly detected masses, even before further confirmations are available. In this way, a monitoring of suspect masses can benefit from the high mass accuracy of DI-FT-ICR MS allowing fast and efficient data processing. The necessity to collaborate on, innovate, and share screening methodologies will not only enhance our understanding of chemical exposure but also create opportunities for wider applications. The refined processes employed in this study, alongside the adaptable *PFlow*, can seamlessly transition to diverse research domains, including the exploration of human exposure, and potentially extend to other emerging compound classes beyond PFAS.

### Supplementary Information

Below is the link to the electronic supplementary material.

Supplementary file1 (DOCX 10404 KB)

Supplementary file2 (KNWF 272 KB)

Supplementary file3 (XLSX 601 KB)

## Data Availability

The *PFAS:SL* and *PFlow* workflows can be downloaded from 10.5281/zenodo.11633375. KNIME Analytics Platform (version 4.7.5) is required to run the workflows.
