# Supplementary material for: An automated and high-throughput data processing workflow for PFAS identification in biota by direct infusion ultra-high resolution mass spectrometry
Source: Anal Bioanal Chem. 2024 Aug 1;416(22):4833–48. doi: 10.1007/s00216-024-05426-2 (PMC11330400; doi:10.1007/s00216-024-05426-2)
Supplement: Supplementary file 1 — Supplementary file1 (DOCX 10404 KB) [file 216_2024_5426_MOESM1_ESM.docx]

**Supporting Information**

# **An automated and high-throughput data processing workflow for PFAS identification in biota by direct infusion ultrahigh resolution mass spectrometry.**

Silvia Dudášová^1^, Johann Wurz^1^, Urs Berger^1,$^, Thorsten Reemtsma^1,2^, Qiuguo Fu^1,*^, Oliver J. Lechtenfeld^1,3,*^

^1^ Department of Environmental Analytical Chemistry, Helmholtz Centre for Environmental Research - UFZ, Permoserstraße 15, 04318 Leipzig, Germany.

^2^ Institute for Analytical Chemistry, University of Leipzig, Linnéstrasse 3, 04103 Leipzig, Germany.

^3^ ProVIS - Centre for Chemical Microscopy, Helmholtz Centre for Environmental Research - UFZ, Permoserstraße 15, 04318 Leipzig, Germany.

^$^ present address: Laboratory of Clinical Biochemistry and Metabolism, Department of General Pediatrics, Adolescent Medicine and Neonatology, Faculty of Medicine, University of Freiburg, 79106 Freiburg, Germany

*Corresponding to:

Helmholtz-Centre for Environmental Research – UFZ

Department of Environmental Analytical Chemistry

Permoserstrasse 15, 04318 Leipzig, Germany

Dr. Oliver Lechtenfeld and Dr. Qiuguo Fu

E-mail address: oliver.lechtenfeld@ufz.de; qiuguo.fu@ufz.de

# **S1. Suspect list: PFAS:SL; documentation and notes.**

- Blue_obelisk_repository.csv: Mass recalculation
- CompTox Chemicals Dashboard v2.3.0: “*PFAS Master List of PFAS Substances*”, Last update: 2023-02-27, Downloaded: 2022-10-10, Number of entries: 12,034 (Downloaded from: <https://comptox.epa.gov/dashboard/chemical-lists/PFASMASTER>)

## **S2 A. Generation of the suspect list**

**Configuration.** PFAS CompTox list serves as a foundation for our suspect list; therefore, it was selected as the input file with *the Blue Obelisk Repository element mass list* used for monoisotopic mass recalculation and isotope mass calculation in the molecular parameters’ component.

**Data cleaning.** The KNIME manipulation nodes and the RDKit nodes were used to retrieve information on the IUPAC (International Union of Pure and Applied Chemistry) name either from the “preferred name” column or InChI Key (International Chemical Identifier). Secondly, InChI key was recovered from the existing IUPAC name following MF generation that was parsed to a JSON data string with custom Python code. Entries with the common IUPAC name, InChI key and molecular formula were removed.

**Molecular parameters recalculation.** All calculations of chemical molecular parameters and isotopologues of the selected isotopes were calculated by using KNIME manipulation nodes and Python scripts. All monoisotopic masses were recalculated based on *the Blue Obelisk repository element list.*

**Output directory.** Results were written in the chosen output directory within the KNIME workspace in the folder named with the current date in the following format: yyyy-MM-dd.


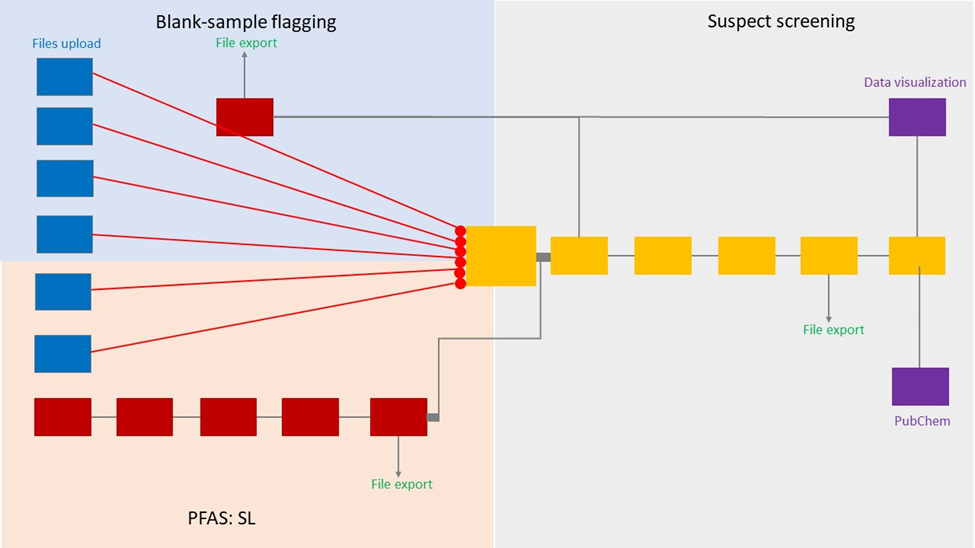


**Figure S1.** The schematic diagram represents the multifunctional capabilities of our workflow, delineated by three primary colors each signifying a distinct task: a.) Blank-Sample Flagging (blue): This phase involves the comparative analysis between blank and sample datasets to identify and flag common masses. b.) PFAS Suspect List Creation (orange): This step focuses on the generation of a comprehensive list of PFAS suspects, and c.) PFAS Suspect Screening (grey): In this final stage, the workflow systematically screens the suspect list against experimental data. It employs sophisticated matching algorithms and validation techniques to identify PFAS compounds present in the samples.

**
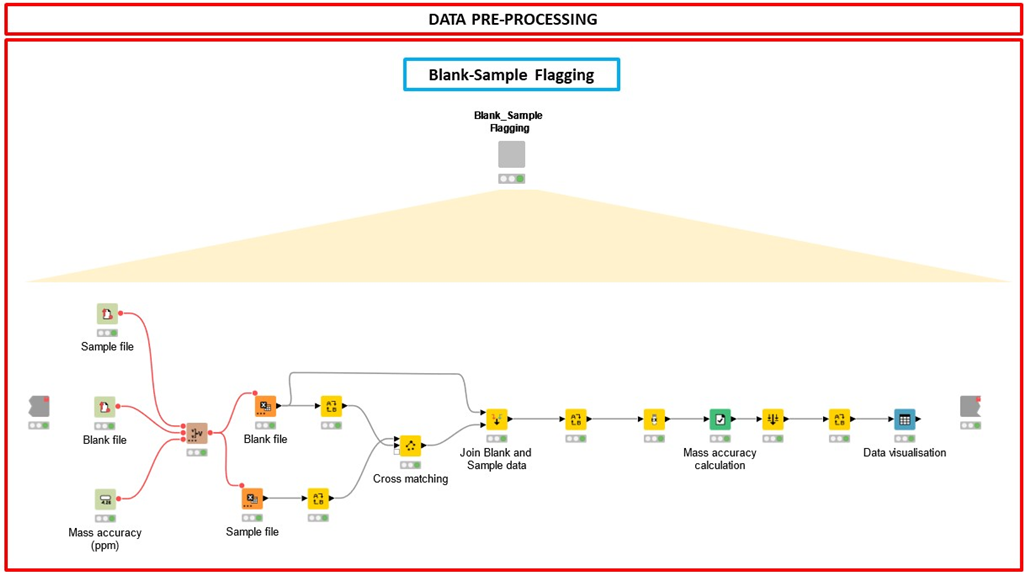
**

**Figure S1A.** Blank-sample flagging involves comparing data from sample and blank tests to identify common masses within a specified mass accuracy range, measured in parts per million (ppm). When signals for these common masses are detected, the masses are "flagged" to indicate the presence and ratio of these signals between the sample and the blank. This process helps in distinguishing relevant analytical signals from potential contaminants or background noise.

**
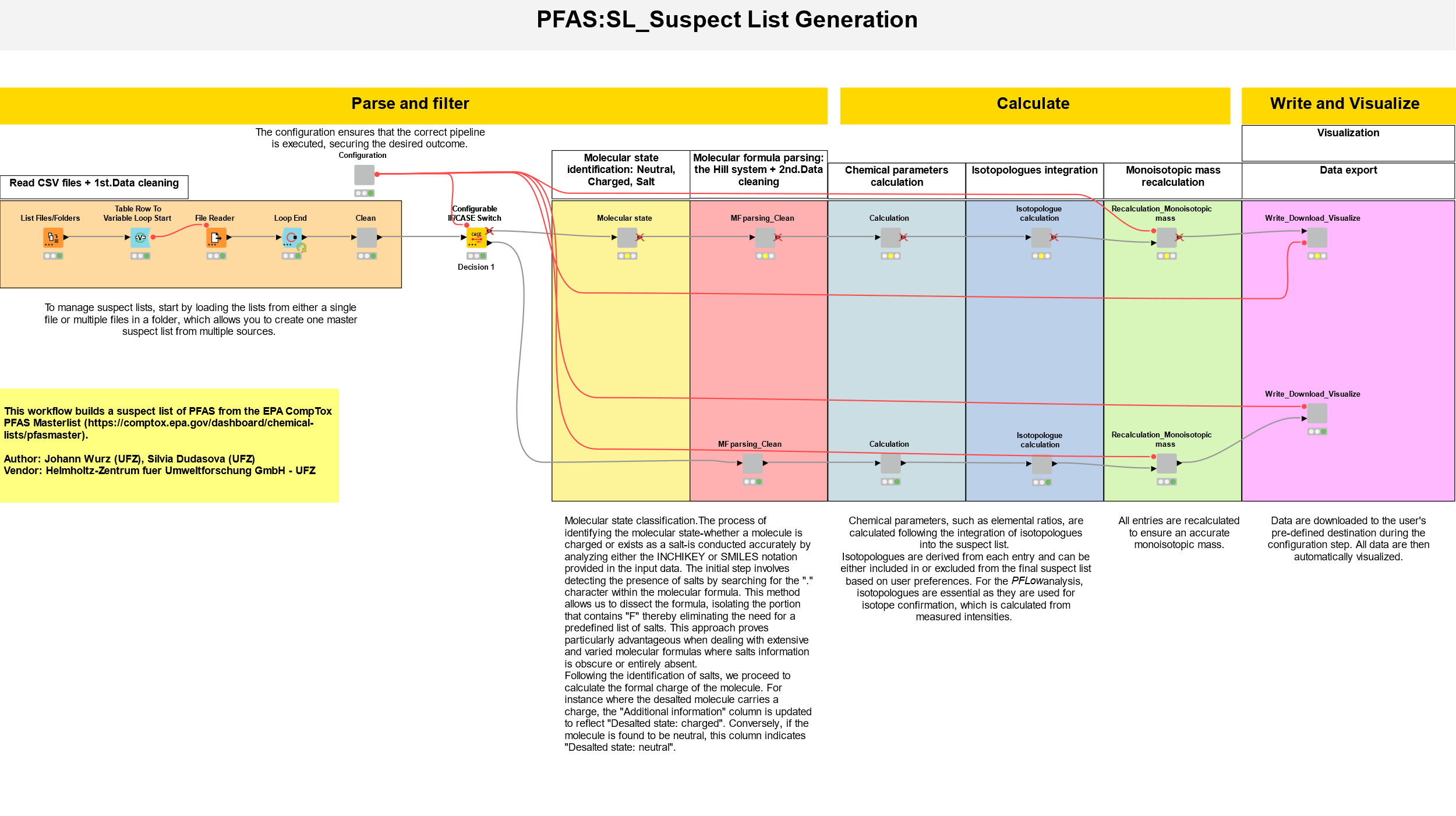
**

**Figure S1B.** The workflow for creating suspect lists is a structured process that begins with data upload, where users can input their data into the system. To ensure data integrity and uniqueness, the workflow includes a step for removing duplicates based on INCHIKEY, MOLECULAR FORMULA, and MONOISOTOPIC MASS. This is crucial as each row in the table must contain at least one piece of data from these columns; rows lacking data in all three are excluded.

A critical step involves removing entries missing the “MONOISOTOPIC MASS” as this indicates that the provided data represents only a portion of the molecule, which can compromise the accuracy of subsequent analyses. We also incorporate a function to reorder elements according to the Hill system, enhancing the consistency and readability of molecular formulas.

Isotope removal from the list is another important step, followed by the "Molecular State" classification. In our workflow, the process of identifying the molecular state—whether a molecule is charged or exists as a salt—is conducted accurately by analyzing either the (International Chemical Identifier) InChI string or SMILES (Simplified Molecular Input Line Entry System) notation provided in the input table. The initial step involves detecting the presence of salts by searching for the "." character within the molecular formula. This method allows us to dissect the formula, isolating the portion that contains "F," thereby eliminating the need for a predefined list of salts. This approach proves particularly advantageous when dealing with extensive and varied molecular formulas where salt information is obscure or entirely absent. Following the identification of salts, we proceed to calculate the formal charge of the molecule. For instances where the desalted molecule carries a charge, the "Additional information" column is updated to reflect "Desalted state: charged." Conversely, if the molecule is found to be neutral, this column indicates "Desalted state: neutral." This systematic sorting extends to molecules initially classified as non-salts; their formal charge is determined, and the "Additional information" column clarifies "Neutral state," while the "Molecular state" explicitly states "Neutral." By categorizing molecules in this detailed manner, our workflow facilitates a more organized and efficient analysis, ensuring each molecule is appropriately classified and treated based on its unique characteristics. This meticulous sorting mechanism enhances our understanding of the molecular landscape within our dataset, enabling targeted and effective analytical study.

The recalculation of monoisotopic mass is conducted directly from the molecular formula. Following this, the workflow progresses to calculate chemical parameters and integrate isotopologues, aiding in the comprehensive analysis and classification of the compounds.

Visualization is the final step in our workflow, allowing users to graphically interpret the data and analyze results. This holistic approach to creating suspect lists streamlines the process of molecular analysis, ensuring accurate and reliable data for researchers.

**
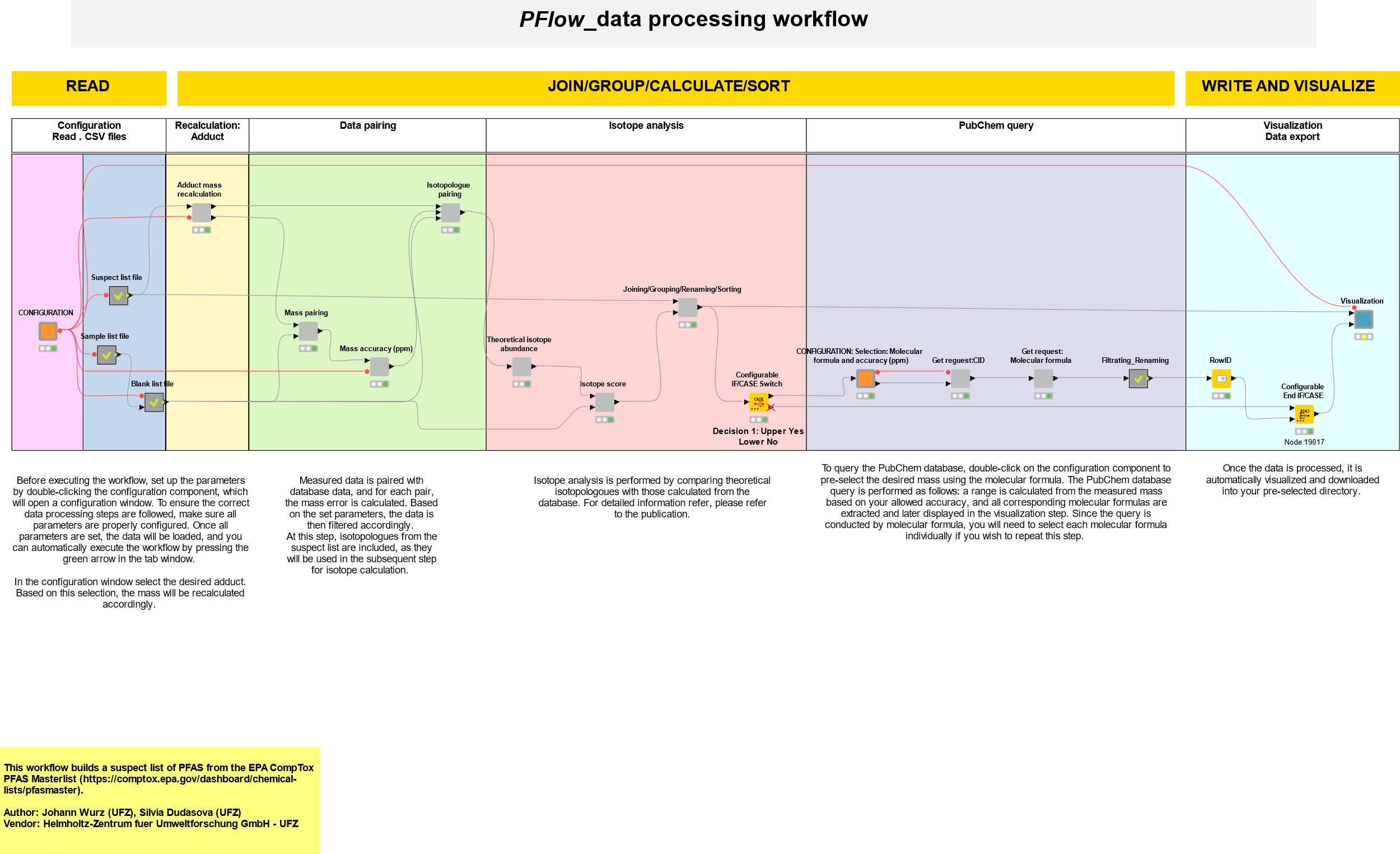
**

**
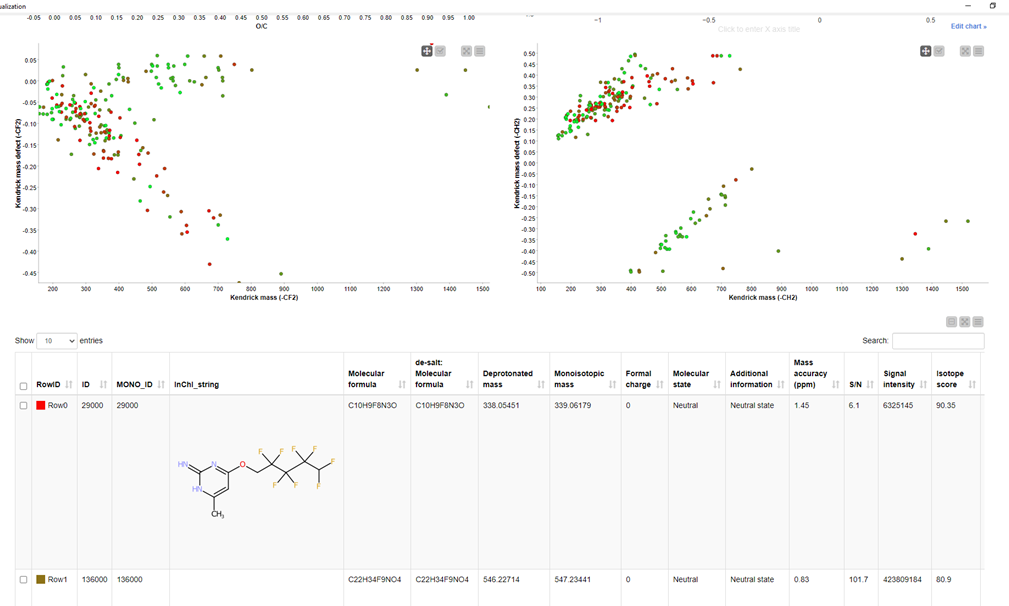
**

**Figure S1C.** The schematic illustration of the suspect screening workflow; *Pflow*. The diagram outlines the workflow's individual components, providing a brief annotation for each and showcasing the sequence of data processing steps leading to the final visualization.


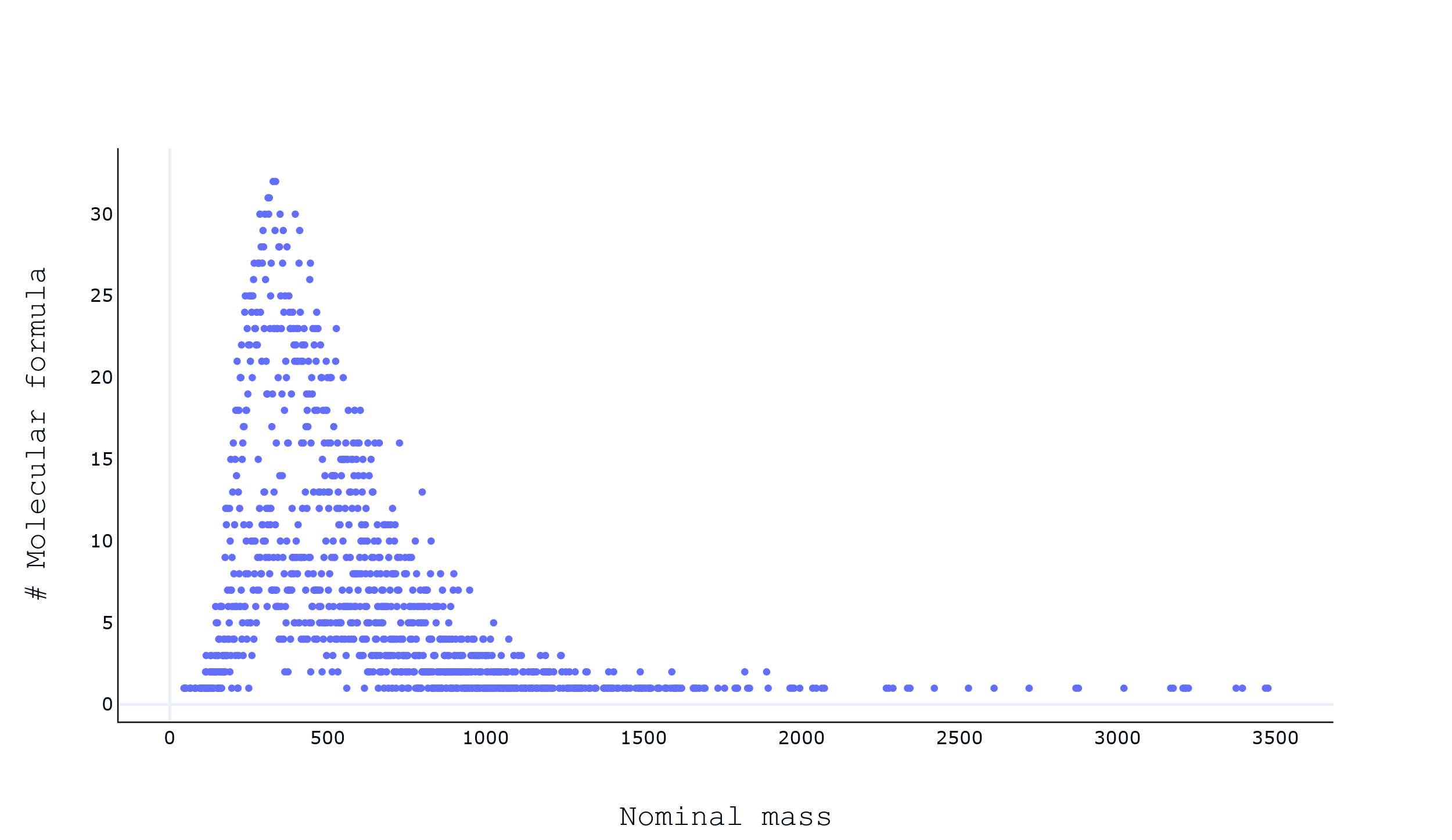


**Figure S2.** Distribution of molecular formulas within the *PFAS:SL*, organized according to their nominal mass. Each point on the graph represents a specific nominal mass value, with the corresponding number of molecular formulas that share the same nominal mass plotted on the y-axis. This visualization illustrates the variability and density of molecular formulas across different mass ranges, highlighting the prevalence of certain nominal masses within the *PFAS:SL*.


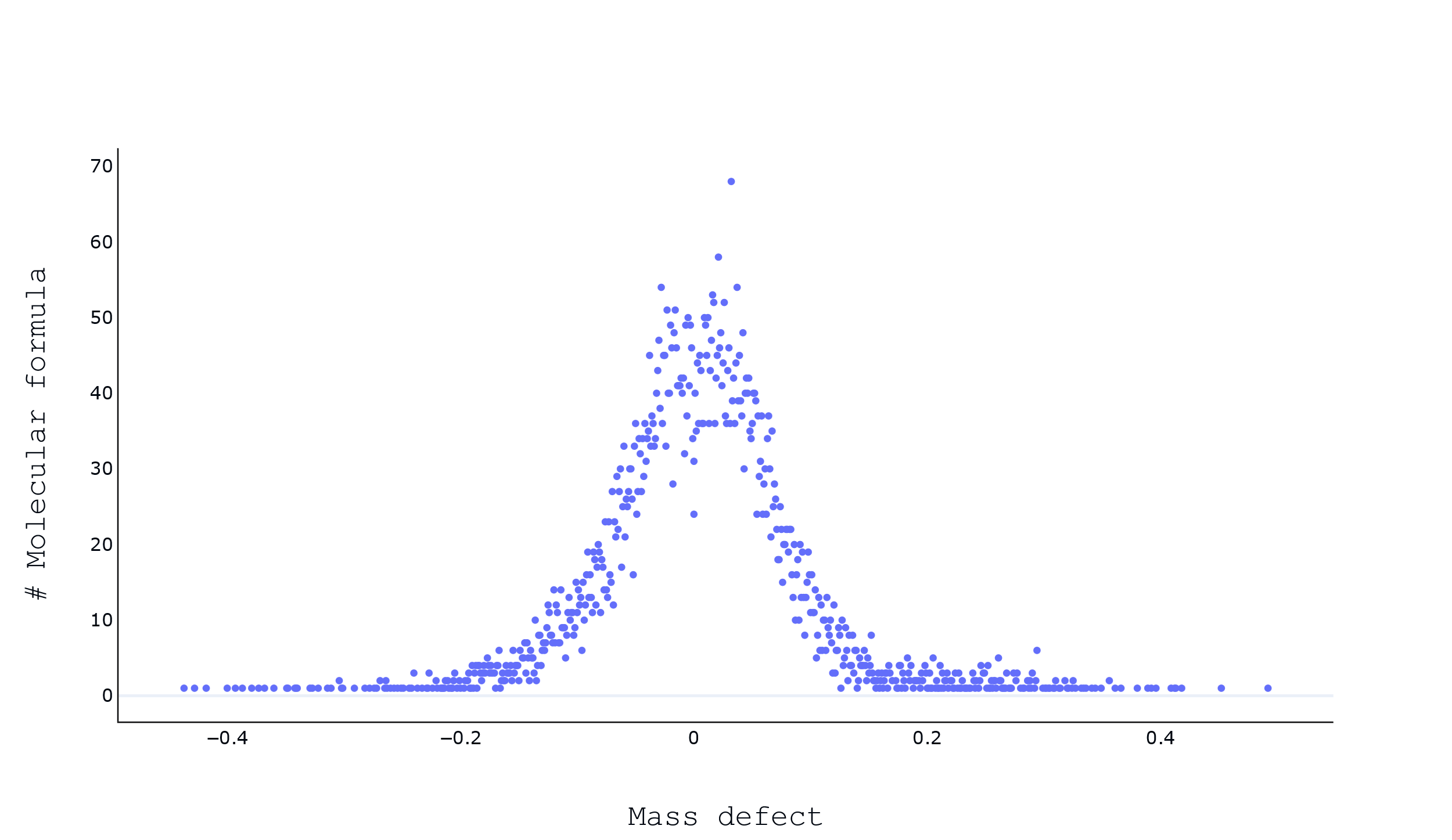


**Figure S3.** Relationship between mass defect and the number of molecular formulas within the *PFAS:SL*, plotted along the x and y axes respectively. A mass defect refers to the difference between the nominal mass and the exact mass of a molecule. The graph reveals a normal distribution pattern, indicating that most molecular formulas cluster around a mass defect value of 0.


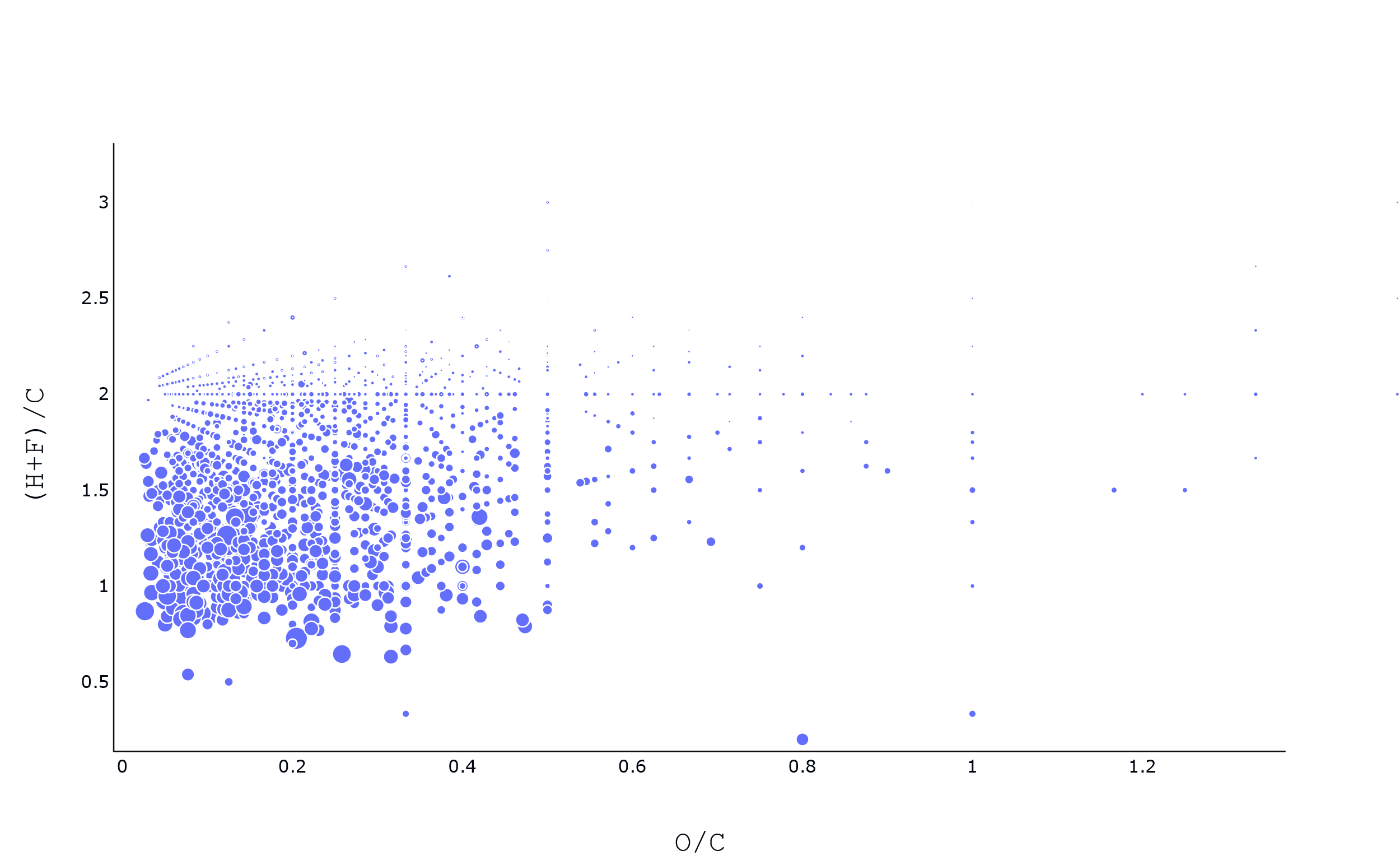


**Figure S4.** Elemental ratio plot, illustrating the ratios of oxygen to carbon (O/C) on the x-axis against the combined ratio of hydrogen and fluorine to carbon (H+F/C) on the y-axis. Each point in the plot represents a molecular formula from the *PFAS:SL*, with the size of the dot varying according to the double bond equivalent (DBE) values of the compounds. Notably, the plot reveals a concentration of larger dots in the lower-left corner, indicating molecular formulas with higher DBE values in this region. This pattern suggests a prevalence of more unsaturated or aromatic compounds within this specific elemental ratio range. Conversely, the rest of the graph features smaller dots, denoting molecular formulas with lower DBE values, which typically correspond to more saturated compounds. This visualization aids in understanding the structural diversity of the compounds within the *PFAS:SL*, highlighting the varying degrees of saturation and the distribution of elemental ratios among the molecular formulas.


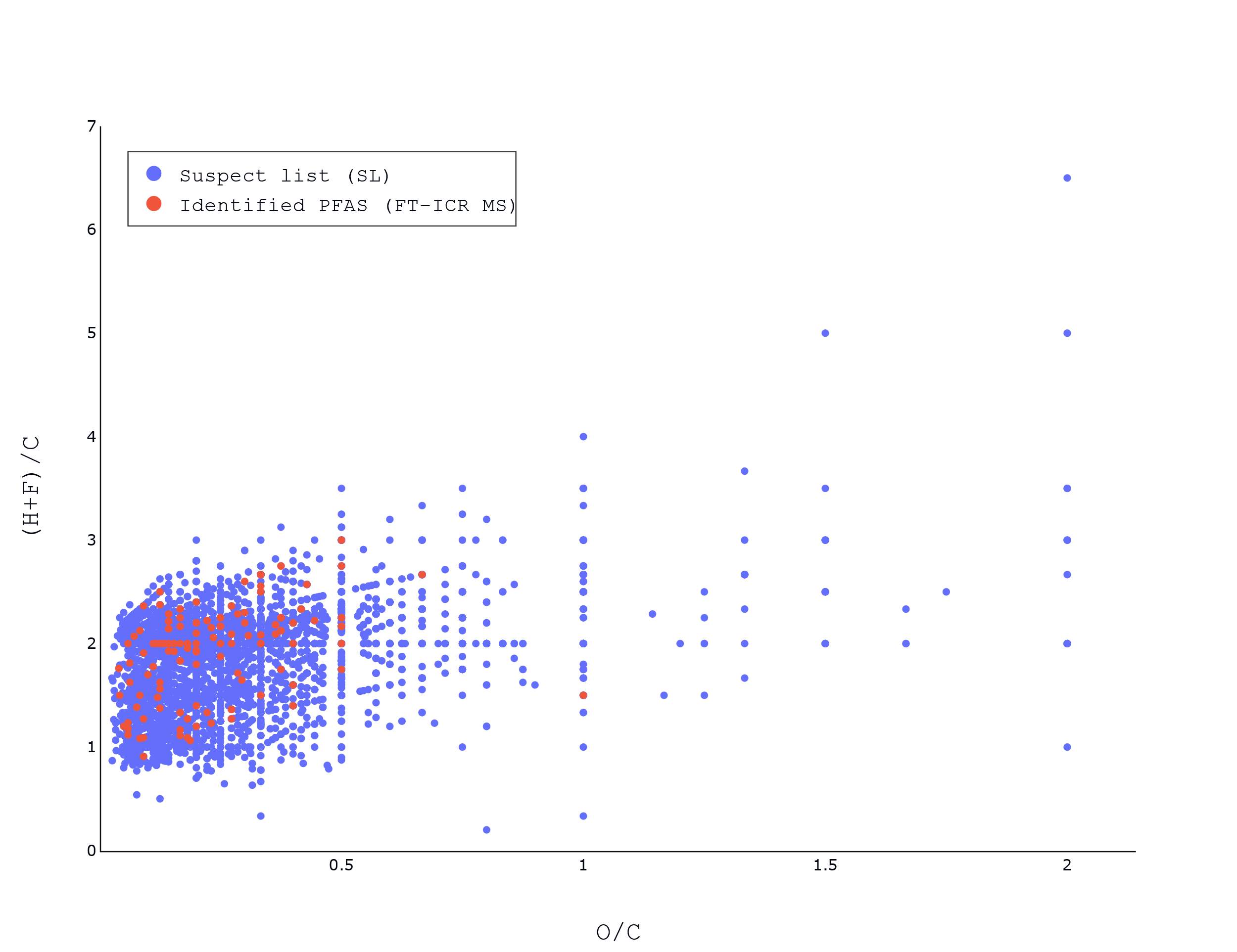


**Figure S5.** This figure presents an elemental ratio plot, graphically illustrating the ratios of oxygen to carbon (O/C) on the x-axis against the combined ratio of hydrogen and fluorine to carbon (H+F/C) on the y-axis. Each point in the plot represents a molecular formula in the *PFAS:SL*. Notably the red dots, representing a total of 84 candidates, illustrate the diversity and distribution of tentatively identified PFAS.

**Figures S6-S14:** The series of figures, spanning from Figure S6 to Figure S14, are systematically structured into three main components. The initial section of each figure showcases a High-resolution Extracted Ion Chromatogram (HR-EIC), which is obtained from full-scan measurements (black outline). Following this, a second trace is introduced in the figures, featuring observed fragments for the respective compound group (red outline). Moreover, each figure incorporates a table displaying supplementary information obtained from *Pflow.* The figures are captioned with the name of the respective group name. Where necessary, an additional text is included in the figure description.
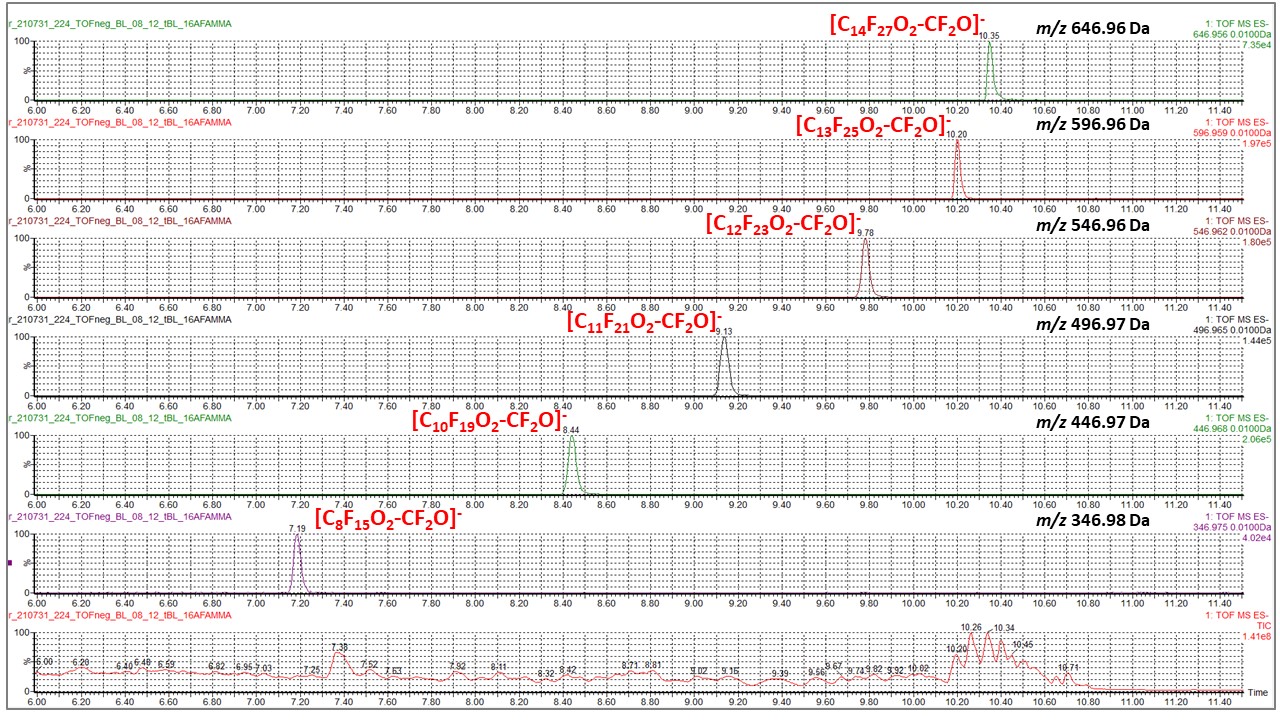


| **Structure** | **Molecular formula** | **[M-H^+^]^-^** | **Rt (min.)** | **Mass accuracy (ppm)** | **Isotope score** | **pKa** | **CASRN** |
| --- | --- | --- | --- | --- | --- | --- | --- |
| 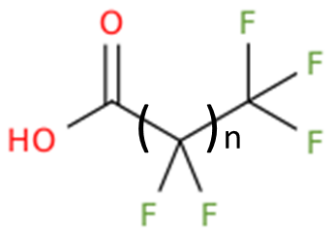 | C_14_HF_27_O_2_ | 712.94726 | 10.35 | 0.58 | - | 0.4 | 376-06-7 |
|  | C_13_HF_25_O_2_ | 662.95046 | 10.2 | 0.72 | 87.7 | 0.4 | 862374-87-6 |
|  | C_12_HF_23_O_2_ | 612.95365 | 9.78 | 0.54 | 88.54 | 0.4 | 307-55-1 |
|  | C_11_HF_21_O_2_ | 562.95684 | 9.13 | 0.42 | 89.39 | 0.4 | 2058-94-8 |
|  | C_10_HF_19_O_2_ | 512.96004 | 8.44 | 0.29 | 90.27 | 0.4 | 73829-36-4 |
|  | C_8_HF_15_O_2_ | 412.96643 | 7.19 | 0.49 | - | 0.3 | 45285-51-6 |

**Figure S6.** The figure showcases a HR-EIC obtained from full-scan measurements, focusing on perfluorocarboxylic acids (PFCAs). It systematically illustrates the elution profiles of six distinct PFCA compounds, arranged according to their sequential order based on elution times. Each of these compounds is identified by its molecular ion minus the -CF_2_OH group. The observed loss of the -CF_2_OH group in M8-PFOA serves as a confirmatory marker for the PFCA group in this analysis; S6: A-D. Accompanying the chromatogram, the figure includes a table that summarizes the data processed by *Pflow*, providing additional information for each identified compound.


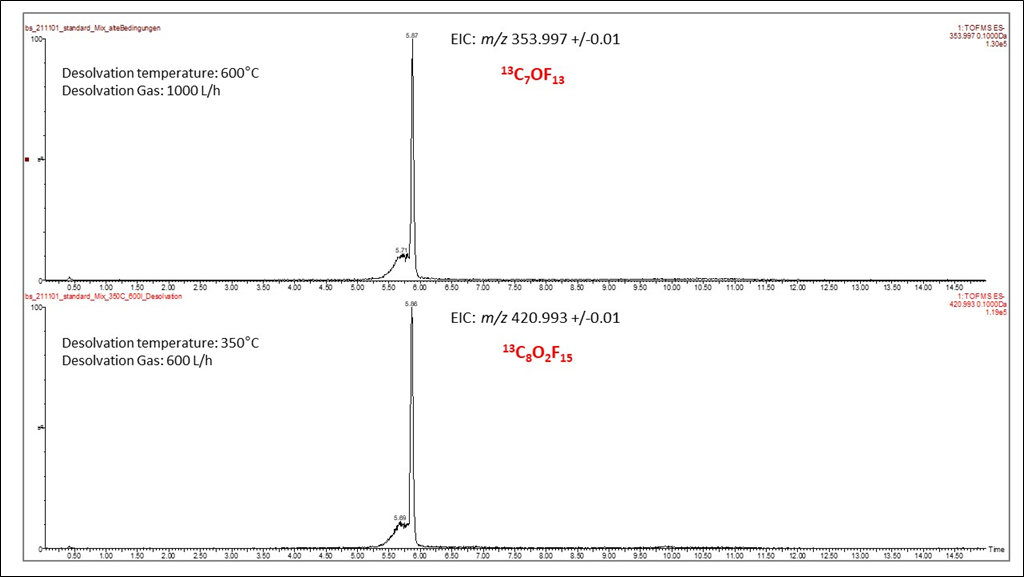


**A.)**

**B.)**


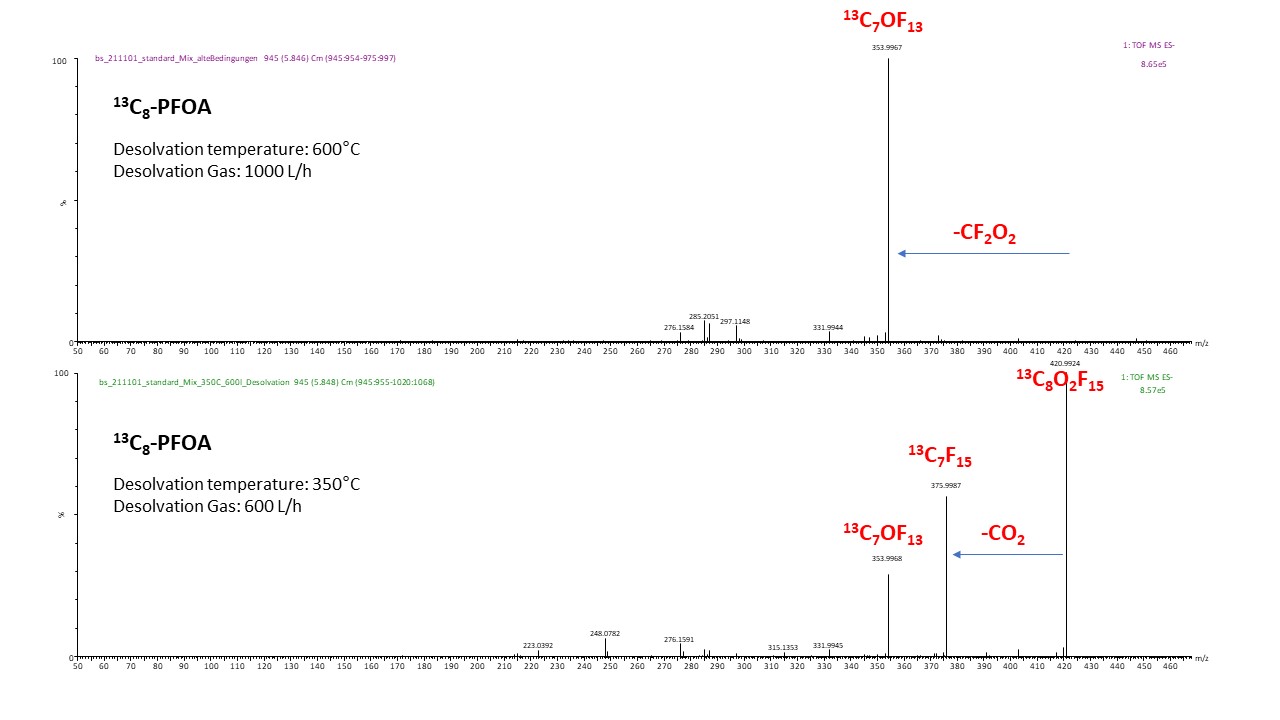


**C.)**

**D.)**

**Figure S6: A-D.** The figure presents a HR-EIC of M8-PFOA, showcasing how different desolvation temperatures impact the observed mass of the molecular ion. Section **A.** of the figure reveals the extractable ion chromatogram for M8-PFOA, with the notable loss of -CF_2_OH, corresponding to an *m/z* of 354 Da. Section **B.** emphasizes the effect of a lower desolvation temperature, leading to the detection of the expected molecular ion at an *m/z* of 421 Da. Furthermore, sections **C.** and **D.** illustrate the mass spectrum, comparing the fragmentation pathway of the molecular ion, specifically highlighting the transition resulting in the loss of -CF_2_OH.


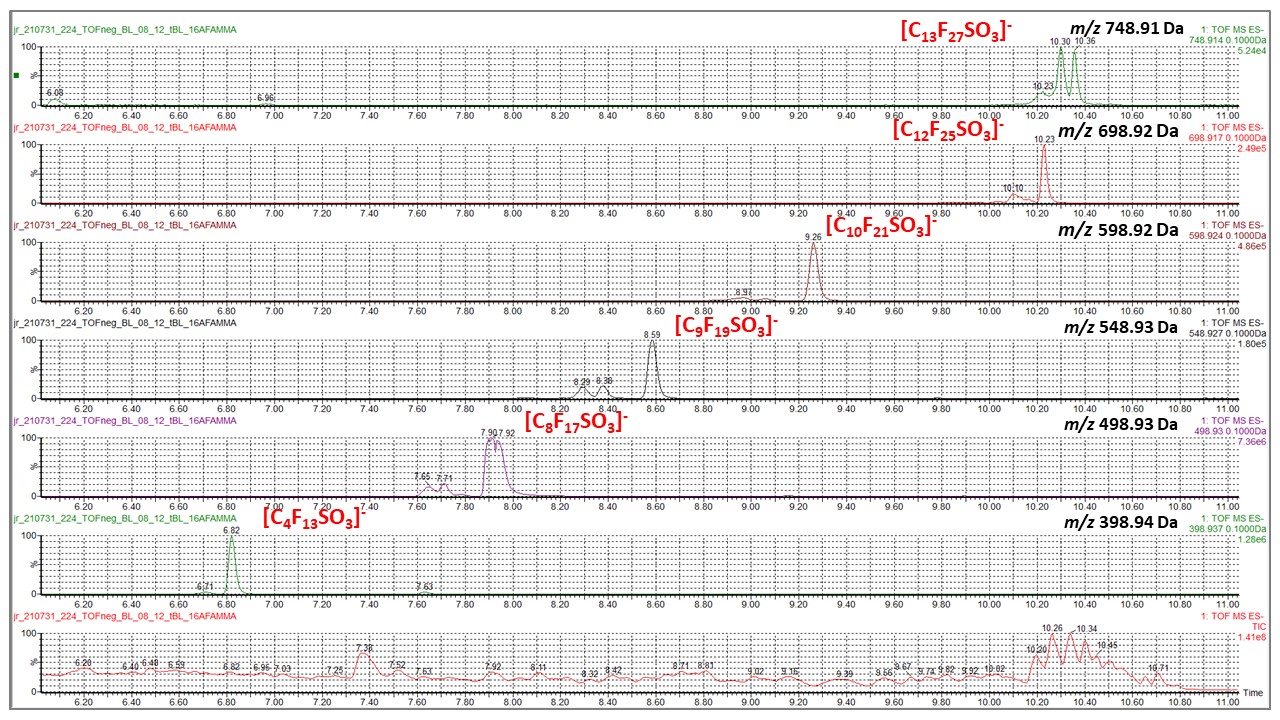


| **Structure** | **Molecular formula** | **[M-H^+^]^-^** | **Rt (min.)** | **Mass accuracy (ppm)** | **Isotope score** | **pKa** | **CASRN** |
| --- | --- | --- | --- | --- | --- | --- | --- |
| 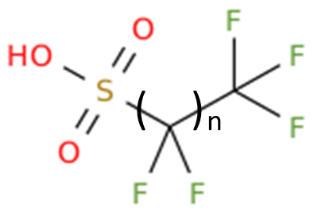 | C_13_HF_27_SO_3_ | 748.91425 | 10.3 | 1.15 | 87.7 | -3.24 | NOCAS_904579 |
|  | C_12_HF_25_SO_3_ | 698.91744 | 10.23 | 0.41 | 88.54 | -3.24 | 79780-39-5 |
|  | C_10_HF_21_SO_3_ | 598.92383 | 9.26 | 0.36 | 86.88 | -3.24 | 126105-34-8 |
|  | C_9_HF_19_SO_3_ | 548.92702 | 8.59 | 0.23 | 91.15 | -3.24 | 68259-12-1 |
|  | C_8_HF_17_SO_3_ | 498.93022 | 7.94 | 0.41 | 88.36 | -3.33 | 255831-20-0 |
|  | C_6_HF_13_SO_3_ | 398.9366 | 6.82 | 0.41 | - | -3.32 | 355-46-4 |

**Figure S7.** The chromatogram illustrates the elution profiles of six homologous perfluoroalkane sulfonic acids (PFSAs), arranged to demonstrate their sequential elution based on an ascending *m/z*. Notably, from 8.25 minutes, the mobile phase transitioned to a 99.9% composed of 2 mM ammonium acetate in a blend of water/methanol/acetonitrile at a ratio of 5/75/20, v/v/v, which led to a distinct narrowing of the elution window specifically between PFDoDS and PFTrDS.


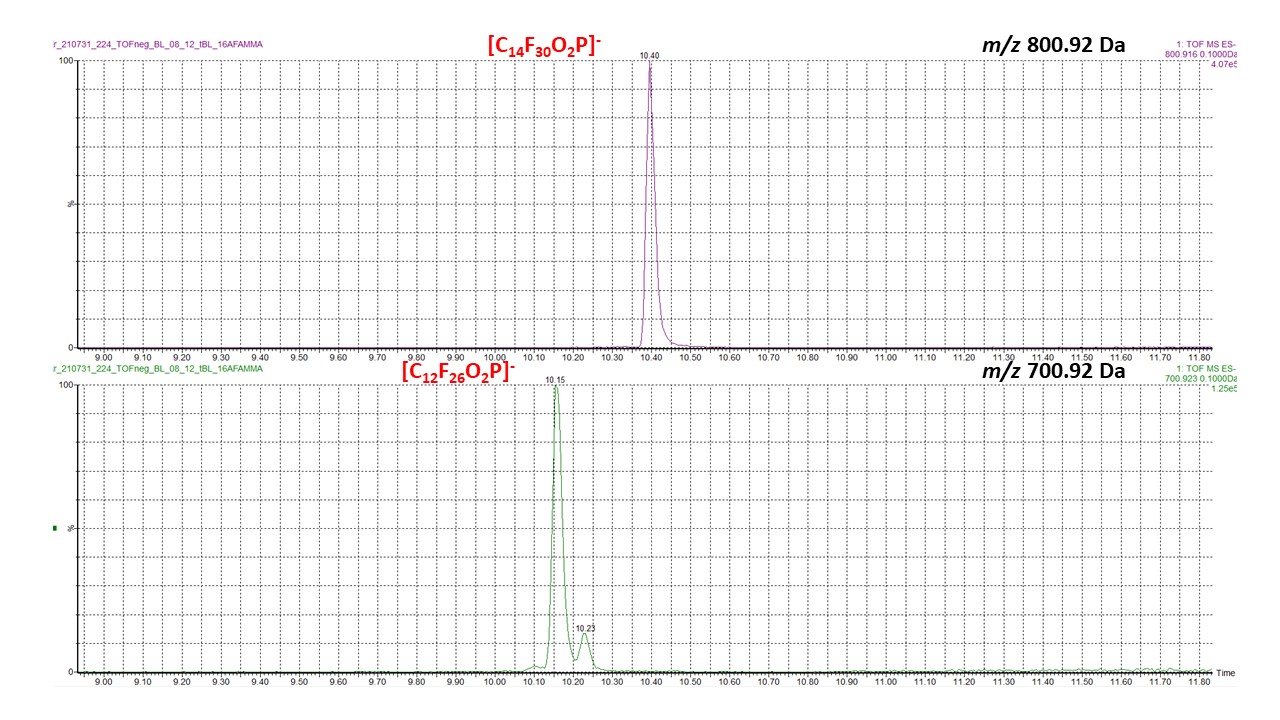

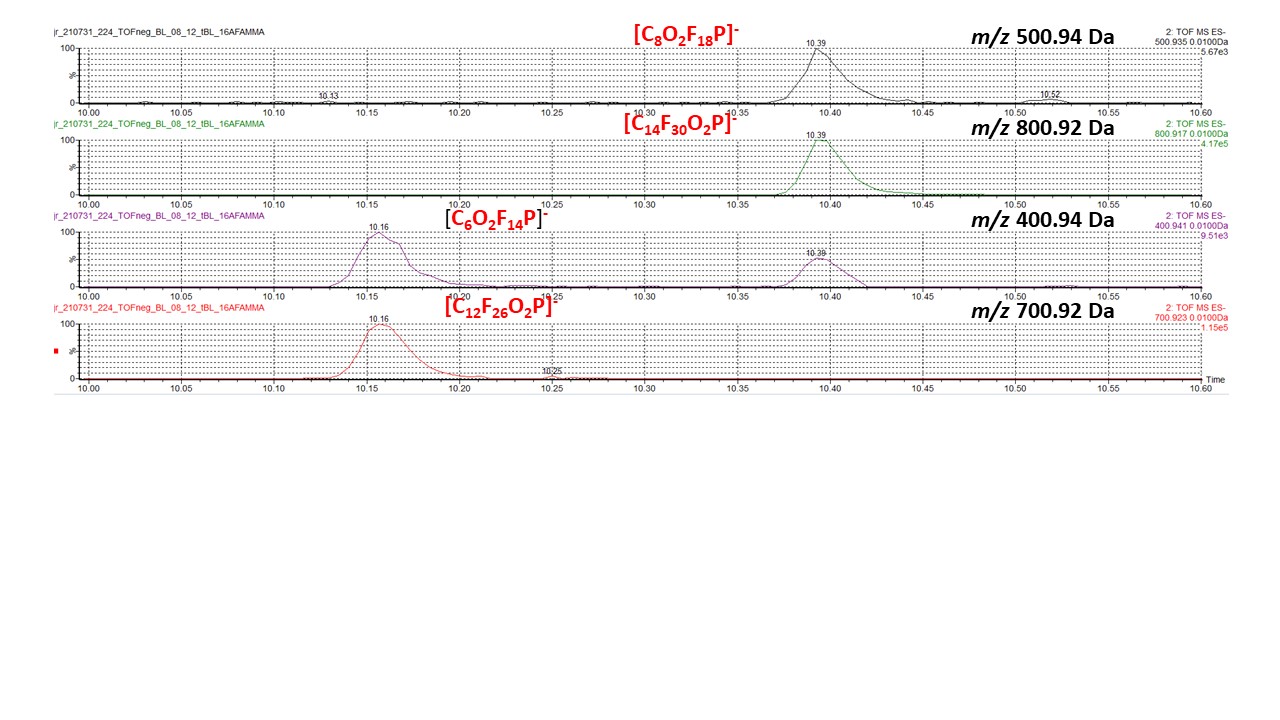


| **Structure** | **Molecular formula** | **[M-H^+^]^-^** | **Rt (min.)** | **Mass accuracy (ppm)** | **Isotope score** | **pKa** | **CASRN** |
| --- | --- | --- | --- | --- | --- | --- | --- |
| 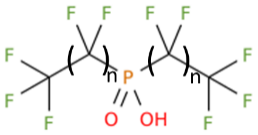 | C_14_HF_30_O_2_P | 800.91624 | 10.4 | 0.78 | 86.88 | 0.09 | 158986-67-5 |
|  | C_12_HF_26_O_2_P | 700.92262 | 10.15 | 0.76 | 88.54 | 0.09 | 610800-35-6 |

**Figure S8.** Perfluorophosphinic acids (PFPiAs); PFPiA (C6/C6) and PFPiA (C6/C8).


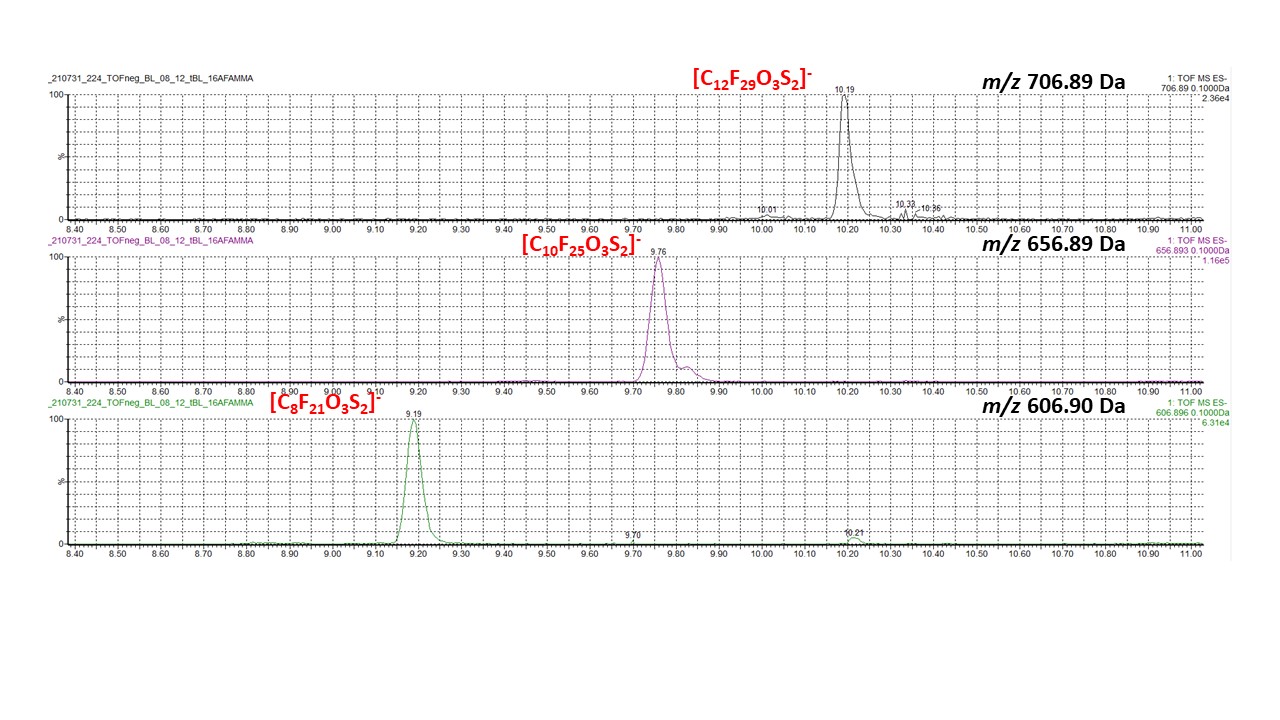

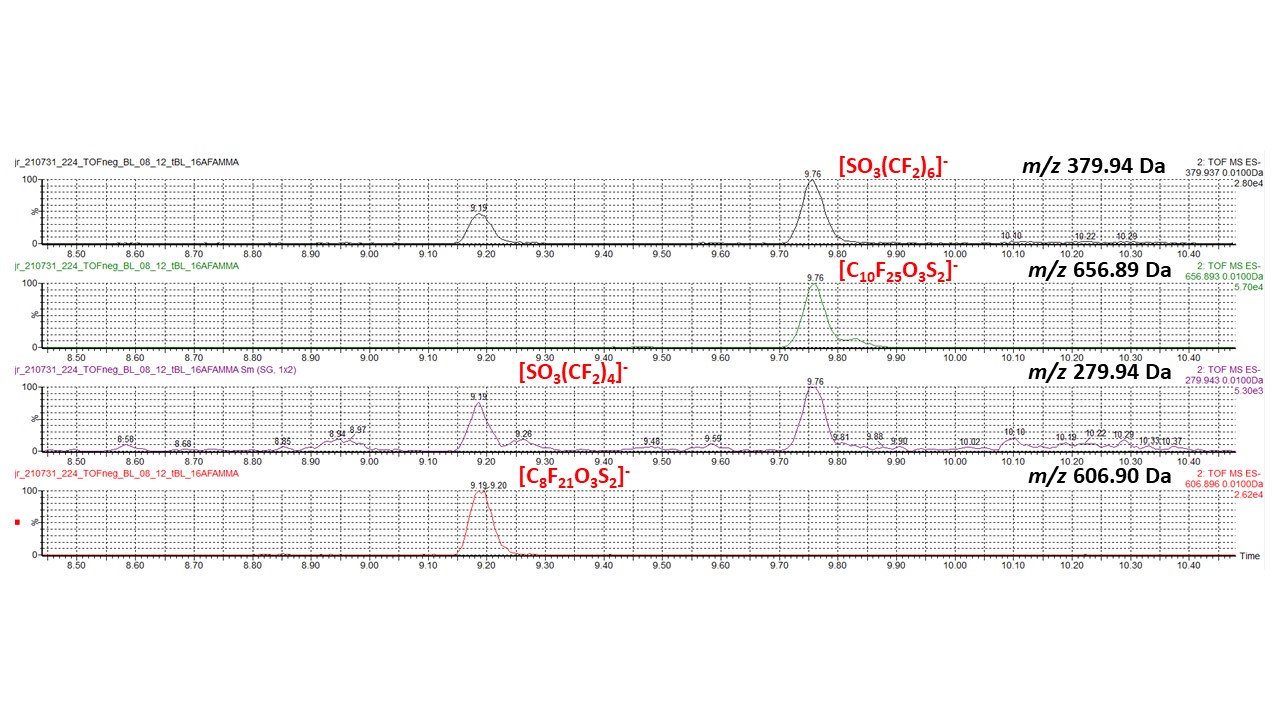


| **Structure** | **Molecular formula** | **[M-H^+^]^-^** | **Rt (min.)** | **Mass accuracy (ppm)** | **Isotope score** | **pKa** | **CASRN** |
| --- | --- | --- | --- | --- | --- | --- | --- |
| 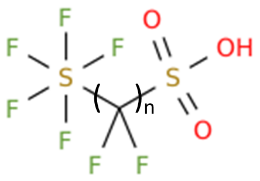 | C_10_HF_25_O_3_S_2_ | 706.88951 | 10.19 | 0.84 | - | -3.24 | NOCAS_1032243 |
|  | C_9_HF_23_O_3_S_2_ | 656.89271 | 9.76 | 0.7 | 87.58 | -3.24 | 2089109-35-1 |
|  | C_8_HF_21_O_3_S_2_ | 606.8959 | 9.19 | 0.33 | - | -3.27 | 2089109-34-0 |

**Figure S9.**  Pentafluorosulfanyl-sulfonic acids (SF5PFLSA).


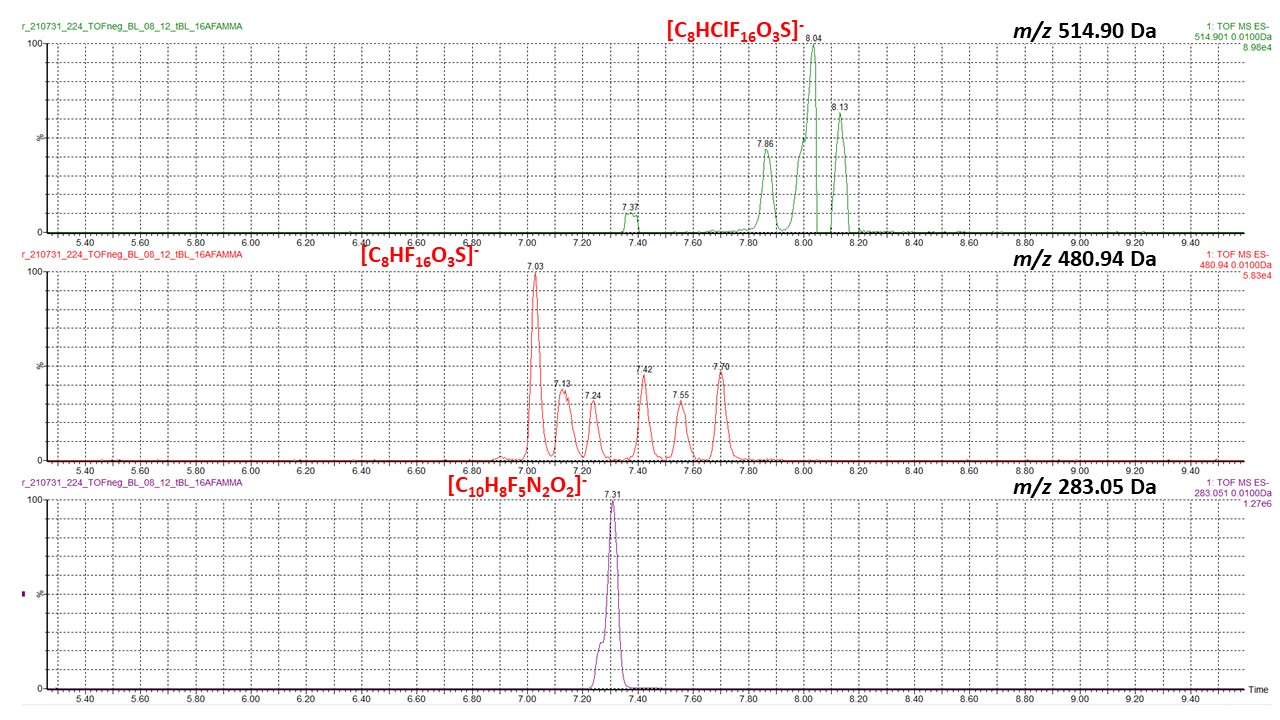


| **Structure** | **Molecular formula** | **[M-H^+^]^-^** | **Rt (min.)** | **Mass accuracy (ppm)** | **Isotope score** | **pKa** | **CASRN** |
| --- | --- | --- | --- | --- | --- | --- | --- |
| 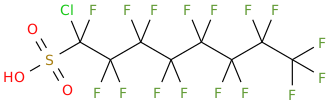 | C_8_HClF_16_O_3_S | 514.90067 | 8.04 | 0.42 | 76.13 | -3.25 | 1651215-26-7 |
| 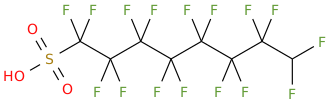 | C_8_H_2_F_16_O_3_S | 480.93964 | 7.7 | 0.85 | - | -3.25 | 134615-57-9 |
| 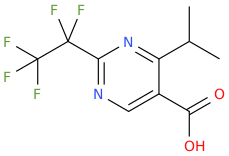 | C_10_H_9_F_5_N_2_O_2_ | 283.05114 | 7.31 | 0.77 | - | 1.88 | 914201-16-4 |

**Figure S10.** The chromatogram features three compounds grouped under perfluoroalkyl acids (PFAAs), specifically highlighting H-PFOS, Cl-PFOS, and a pentafluoroethyl pyrimidine carboxylic acid derivative. Notably, H-PFOS presents with six distinct peaks eluting from 7.03 to 7.90 minutes. This pattern could be indicative of isomeric diversity within the sample or may suggest that the additional peaks result from in-source fragmentation or rearrangement of the “unknown” precursor ions.


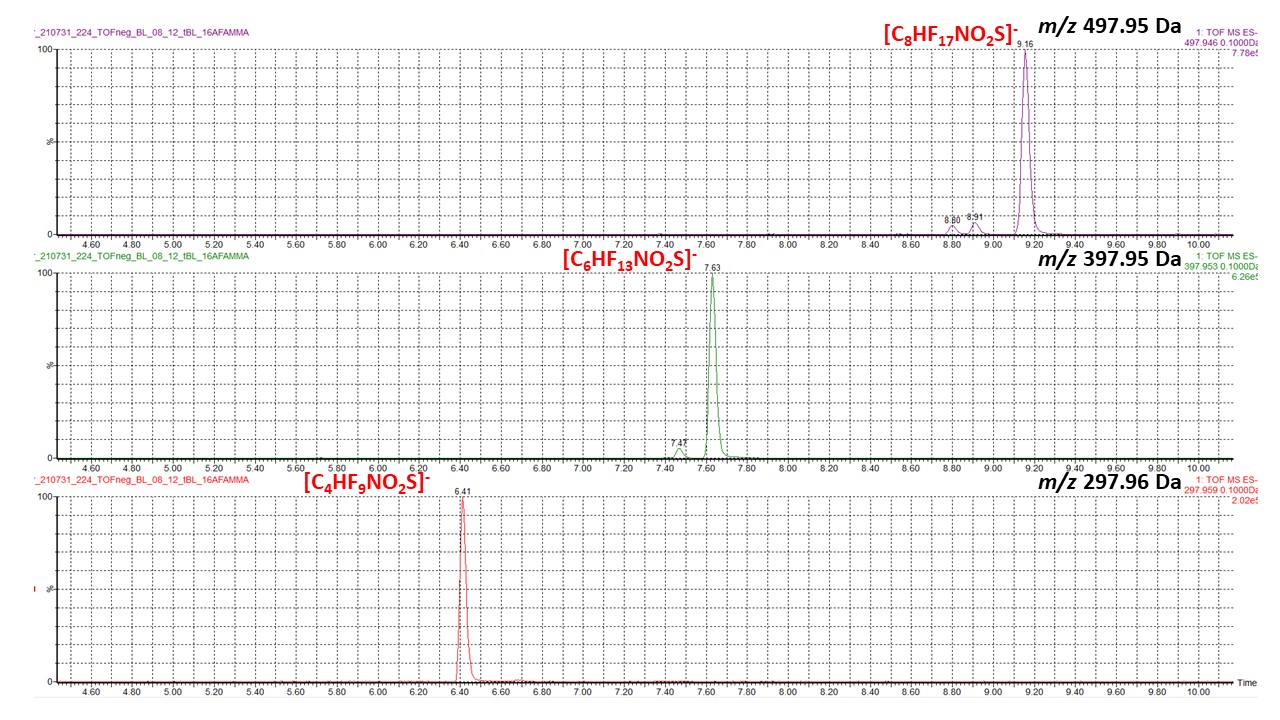

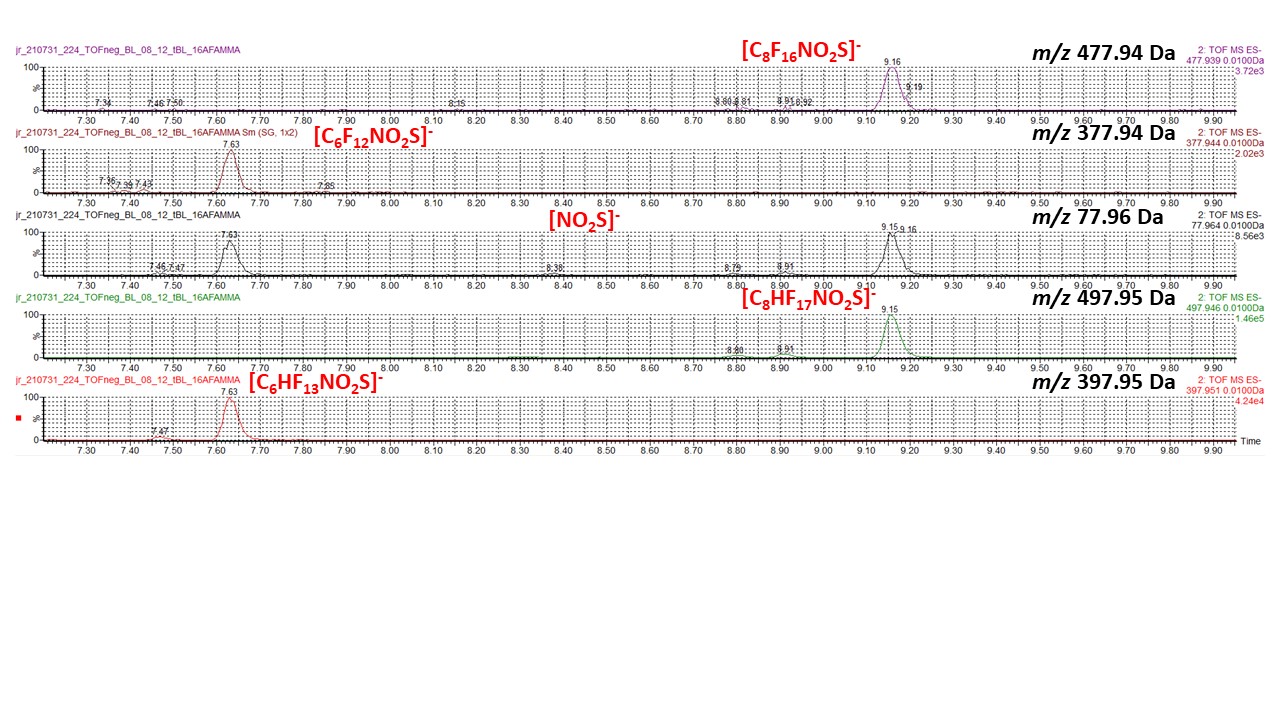


| **Structure** | **Molecular formula** | **[M-H^+^]^-^** | **Rt (min.)** | **Mass accuracy (ppm)** | **Isotope score** | **pKa** | **CASRN** |
| --- | --- | --- | --- | --- | --- | --- | --- |
| 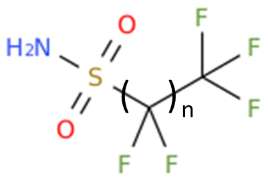 | C_8_H_2_F_17_NO_2_S | 497.9462 | 9.16 | 0.39 | 88.51 | 3.37 | 76752-79-9 |
|  | C_6_H_2_F_13_NO_2_S | 397.95259 | 7.63 | 0.32 | - | 3.37 | 41997-13-1 |
|  | C_4_H_2_F_9_NO_2_S | 297.95898 | 6.41 | 1.23 | - | 3.34 | 30334-69-1 |

**Figure S11.** The Perfluoroalkane sulfonamides (FASA).


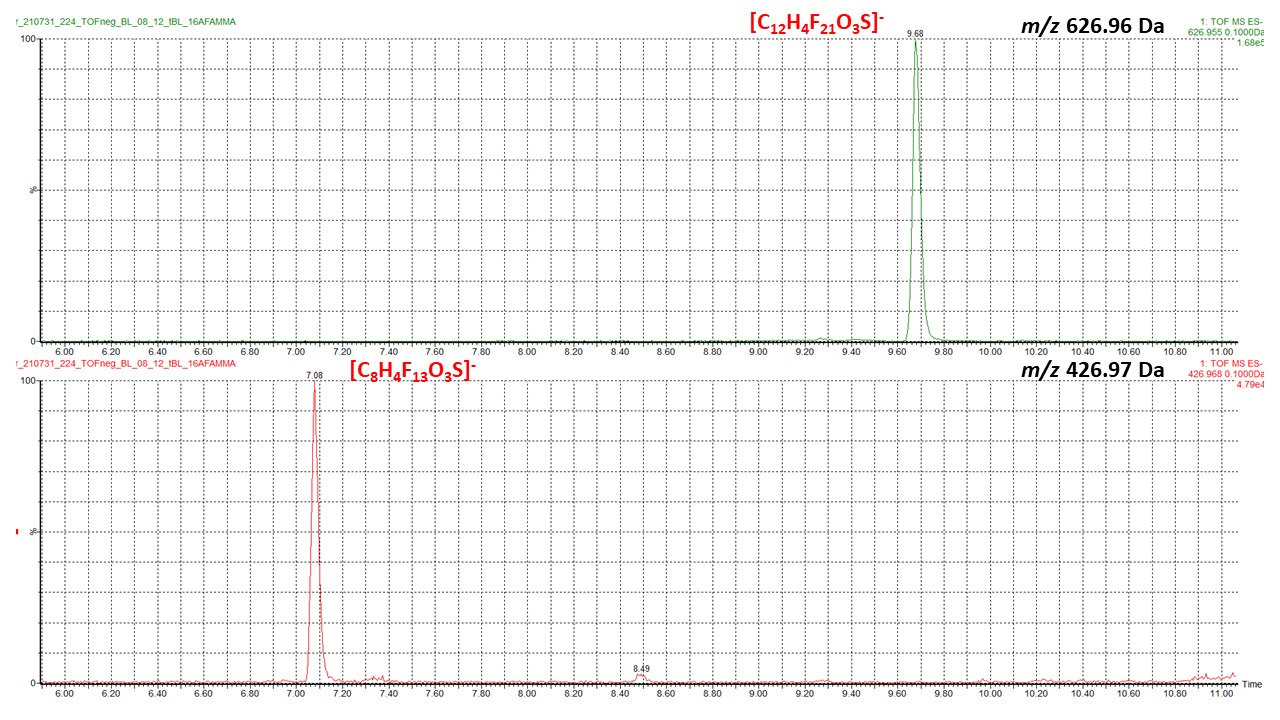

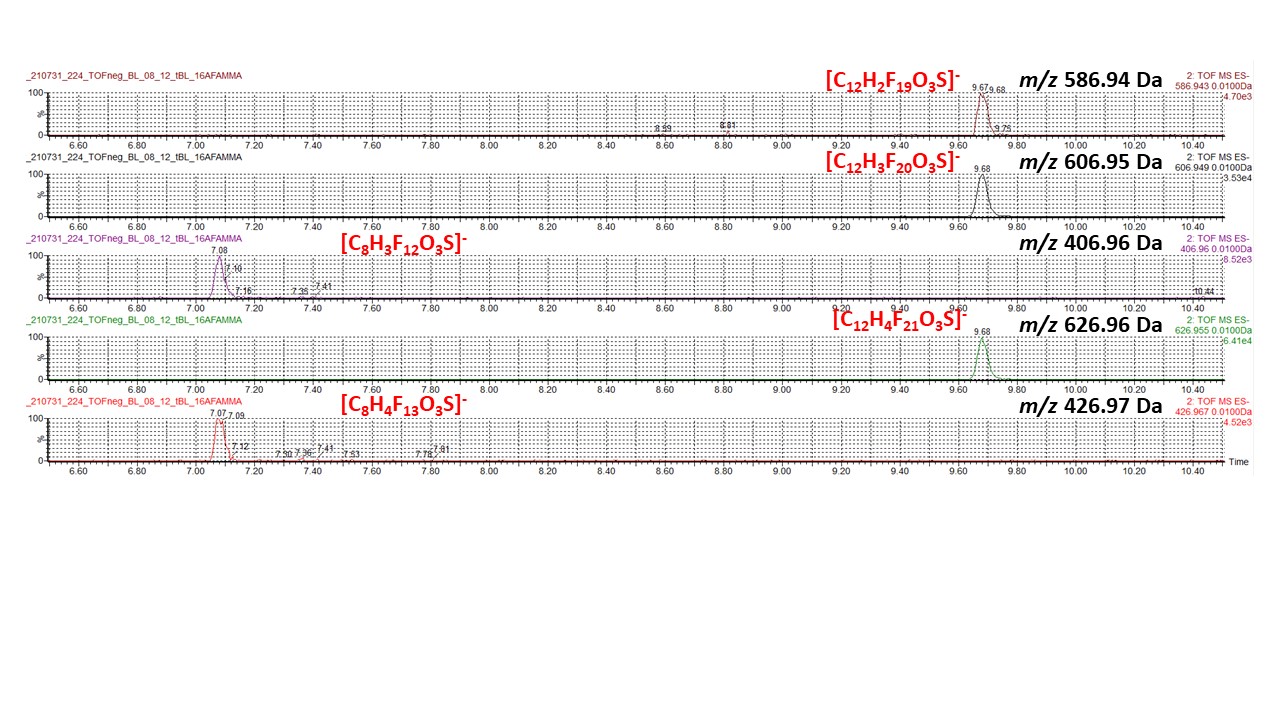


| **Structure** | **Molecular formula** | **[M-H^+^]^-^** | **Rt (min.)** | **Mass accuracy (ppm)** | **Isotope score** | **pKa** | **CASRN** |
| --- | --- | --- | --- | --- | --- | --- | --- |
| 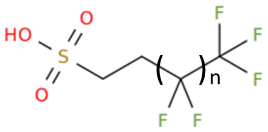 | C_12_H_5_F_21_O_3_S | 626.95513 | 9.68 | 0.46 | - | -2.61 | 120226-60-0 |
|  | C_8_H_5_F_13_O_3_S | 426.9679 | 7.08 | 0.88 | 92.06 | -2.72 | 27619-97-2 |

**Figure S12.** Fluorotelomer sulfonic acids (FTSA).


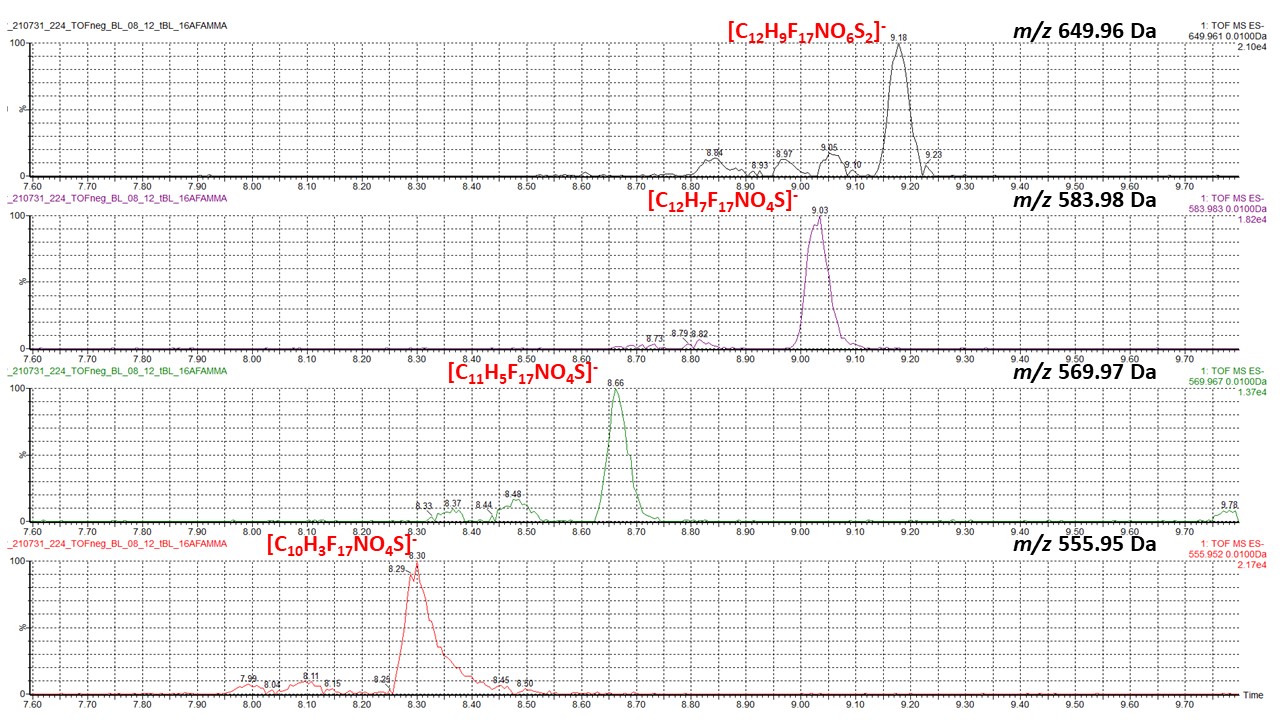
**
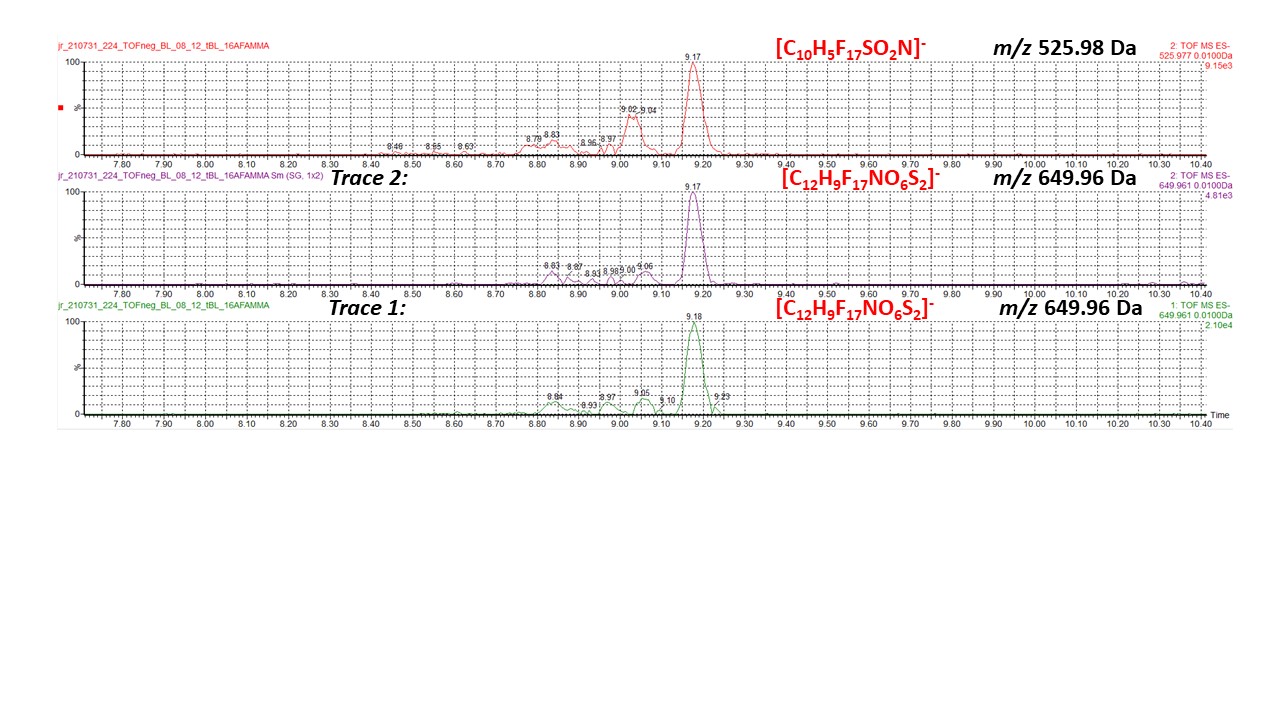
**

| **Structure** | **Molecular formula** | **[M-H^+^]^-^** | **Rt (min.)** | **Mass accuracy (ppm)** | **Isotope score** | **pKa** | **CASRN** |
| --- | --- | --- | --- | --- | --- | --- | --- |
| 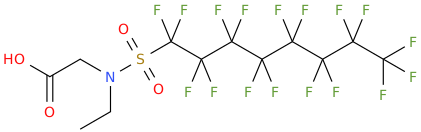 | C_12_H_8_F_17_NO_4_S | 583.98298 | 9.03 | 0.05 | - | 1.71 | 2991-50-6 |
| 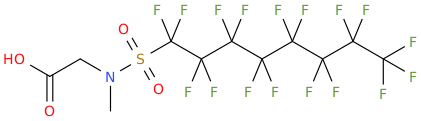 | C_11_H_6_F_17_NO_4_S | 569.96733 | 8.66 | 0.48 | - | 1.6 | 2355-31-9 |
| 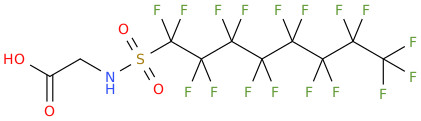 | C_10_H_4_F_17_NO_4_S | 555.95168 | 8.3 | 0.45 | - | 1.45 | 2806-24-8 |
|  |  |  |  |  |  |  |  |
| 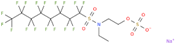 | C_12_H_10_F_17_NO_6_S_2_ | 649.96053 | 9.18 | 0.85 | 88.54 | -2.8 | 2558-75-0 |

**Figure S13.** Perfluorooctane sulfonamido substances with an additional compound named sodium 2-[ethyl(1,1,2,2,3,3,4,4,5,5,6,6,7,7,8,8,8-heptadecafluorooctane-1-sulfonyl)amino]ethyl sulfate.


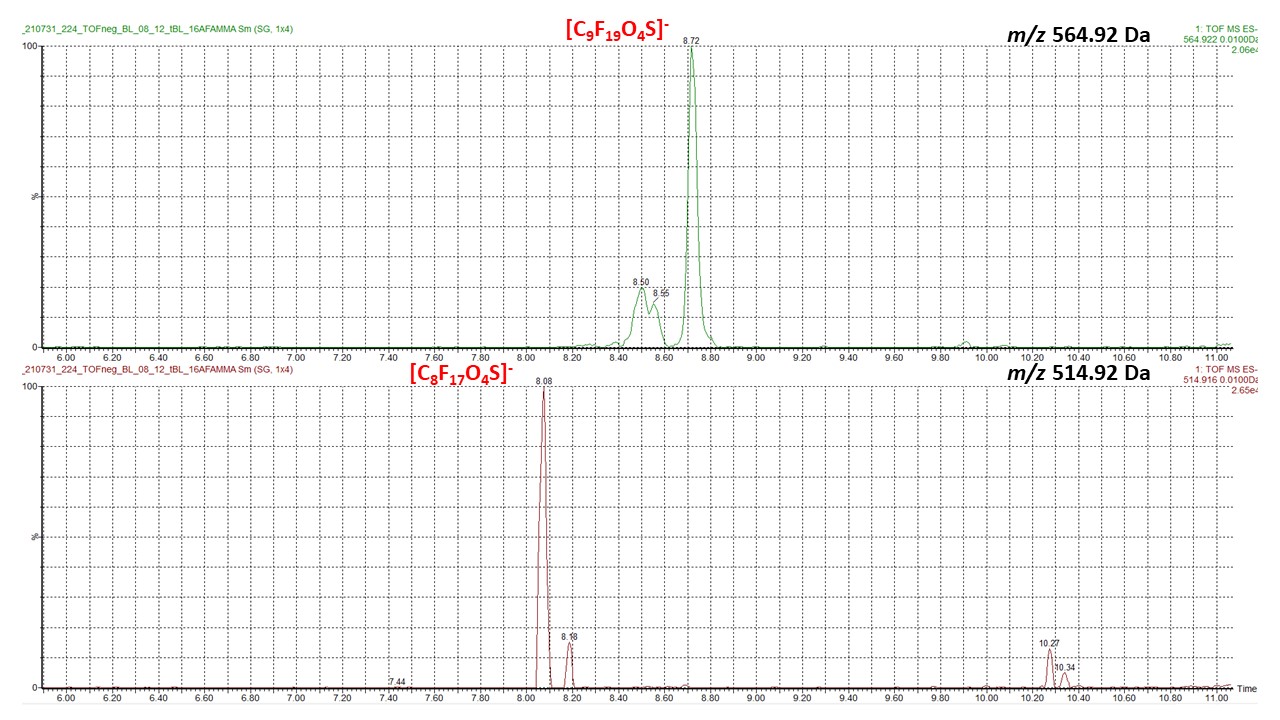

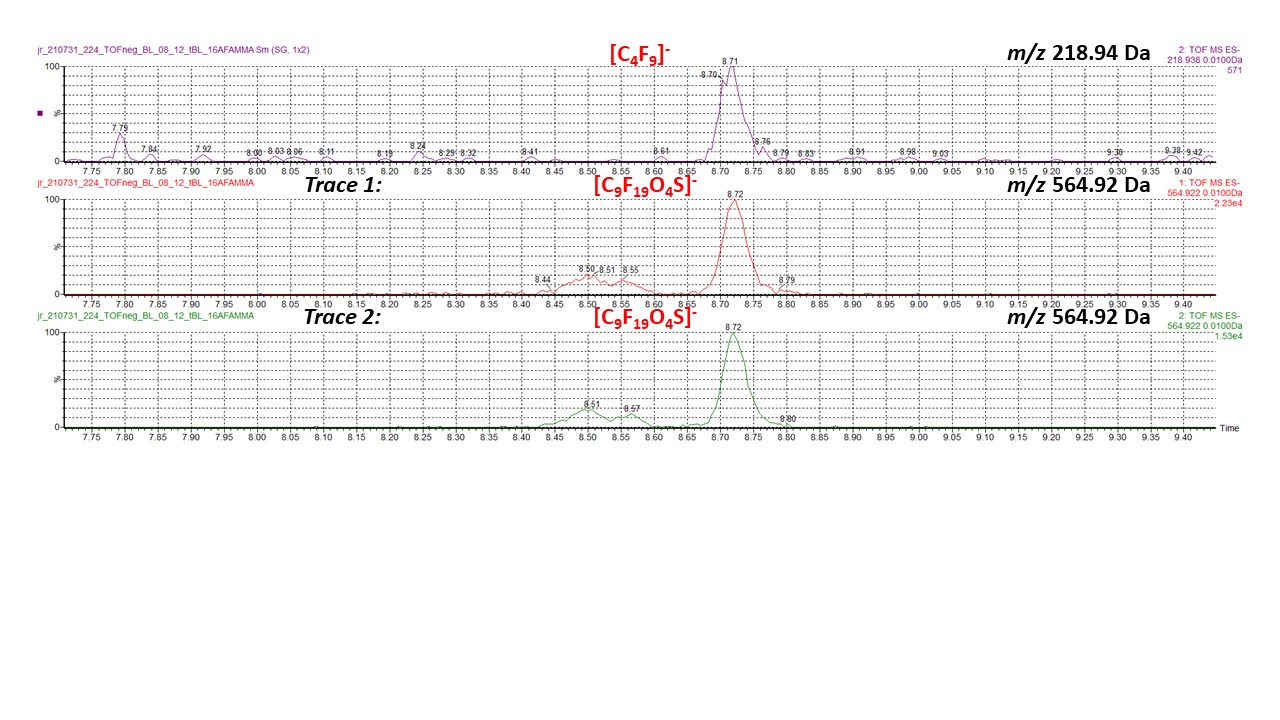


| **Structure** | **Molecular formula** | **[M-H^+^]^-^** | **Rt (min.)** | **Mass accuracy (ppm)** | **Isotope score** | **pKa** | **CASRN** |
| --- | --- | --- | --- | --- | --- | --- | --- |
| 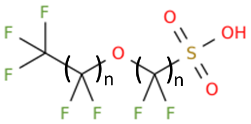 | C_9_HF_19_O_4_S | 564.92194 | 8.72 | 0.36 | - | -3.27 | 914070-83-0 |
|  | C_8_HF_17_O_4_S | 514.92513 | 8.08 | 0.3 | - | -3.25 | 754925-54-7 |

**Figure S14.** Ether-based substances (PFAES).


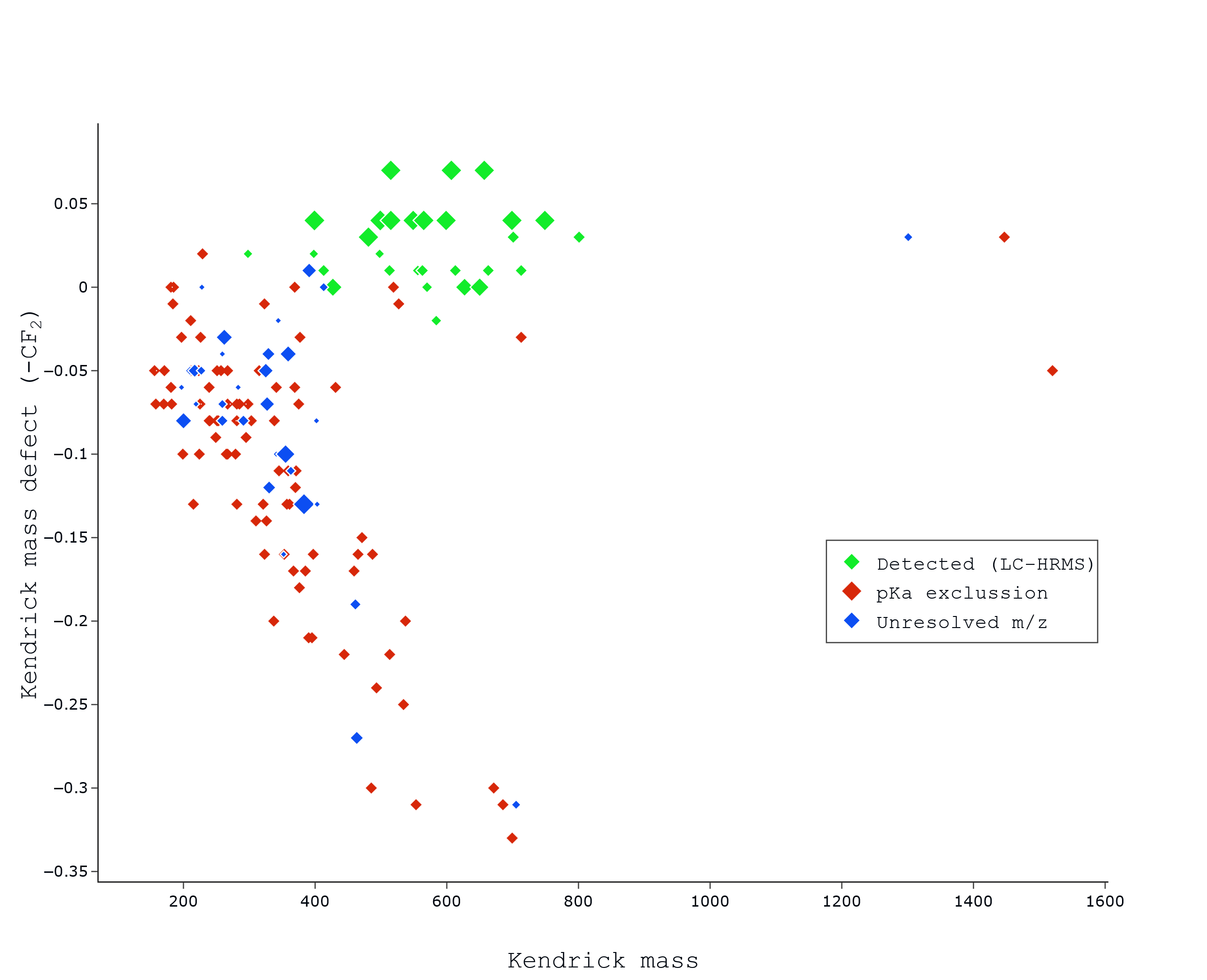


**Figure S15.** Kendrick mass defect plot of candidate PFAS compounds identified by *Pflow*. Candidates are displayed remaining after the initial filtration steps (*n* = 183; cf. Figure 2). The excluded candidates based on the pKa exclusion criterion are represented by red-filled square symbols and compounds detected in LC-HRMS measurements are denoted by green-filled square symbols. Unresolved candidates, which could not be confirmed by LC-HRMS, are depicted with blue-filled square symbols. The size of the “Detected (LC-HRMS)” and “Unresolved m/z)” is inversely proportional to the calculated pKa value. Squares with larger sizes signify lower pKa values, indicating greater acidity. Conversely, smaller squares denote higher pKa values, representing weaker acidity.
